# Truncated acid sphingomyelinase induces substantially accelerated LDL aggregation in vitro

**DOI:** 10.1016/j.jlr.2026.101149

**Published:** 2026-09-15

**Authors:** Alina Iakubovskaia, Akseli Niemelä, Andrea Benković, Andrew House, Arja Pasternack, Pekka Mäntyselkä, Karin Kornmueller, Artturi Koivuniemi, Olli Ritvos, Katariina Öörni

**Affiliations:** 1 Department of Physiology, Faculty of Medicine, University of Helsinki, Helsinki, Finland; 2 Molecular and Integrative Biological Sciences, Faculty of Biological and Environmental Sciences, University of Helsinki, Helsinki, Finland; 3 Atherosclerosis Research Laboratory, Wihuri Research Institute, Helsinki, Finland; 4 Division of Pharmaceutical Biosciences, Faculty of Pharmacy, University of Helsinki, Helsinki, Finland; 5 Gottfried Schatz Research Center for Cell Signaling, Metabolism and Aging, Medical Physics and Biophysics Division, Medical University of Graz, Graz, Austria; 6 Helsinki University Lipidomics Unit (HiLIPID), Helsinki Institute of Life Science (HiLIFE), Biocenter Finland, University of Helsinki, Helsinki, Finland; 7 Institute of Public Health and Clinical Nutrition, Faculty of Health Sciences, University of Eastern Finland, Kuopio, Finland, and Teaching Clinic, Wellbeing Services County of North Savo, Kuopio, Finland

**Keywords:** acid sphingomyelinase, atherosclerosis, cardiovascular disease, enzymology, in vitro methods, LDL, LDL aggregation, lipoproteins, molecular dynamics, optofluidic force induction, sphingolipids

## Abstract

High levels of low-density lipoprotein cholesterol (LDL-C) in circulation are linked to an elevated risk of atherosclerotic cardiovascular diseases (ASCVD). LDL contributes to ASCVD by forming plaques under oxidative and lipolytic conditions in the arterial wall. One of the enzymes involved in LDL lipolysis is acid sphingomyelinase (ASMase) found in atherosclerotic plaques. The susceptibility of LDL to aggregate in vitro upon treatment by recombinant human ASMase (rhASMase) has been shown to associate with ASCVD events in patients having established atherosclerosis. We produced three rhASMase variants and compared their activities toward sphingomyelin and LDL particles. The produced rhASMases represent two C-terminally truncated ASMases with His-tags on the opposite termini and a N-terminally tagged full-length version. The truncated rhASMases showed 3.5-fold higher activity on average in all tested conditions than the full-length version, which is consistent with the previous studies on C-terminal cysteine loss. The truncated rhASMases also induced two-fold faster LDL aggregation and showed improved general stability compared to the full-length rhASMase. A novel method for nanoparticle characterization, optofluidic force induction (OF2i), was applied for continuous measurement of the early onset of the LDL aggregate formation, not previously available by alternative methods. Molecular dynamics simulations on the rhASMases exploring potential differences in their lipid surface binding revealed more dynamic nature of the tag-free N-terminus. We also show associations between LDL aggregation susceptibility, sphingomyelin content, and hydrolysis rate, highlighting the role of LDL lipid composition in association with the development of atherosclerosis.

## Graphical abstract

**Figure:**
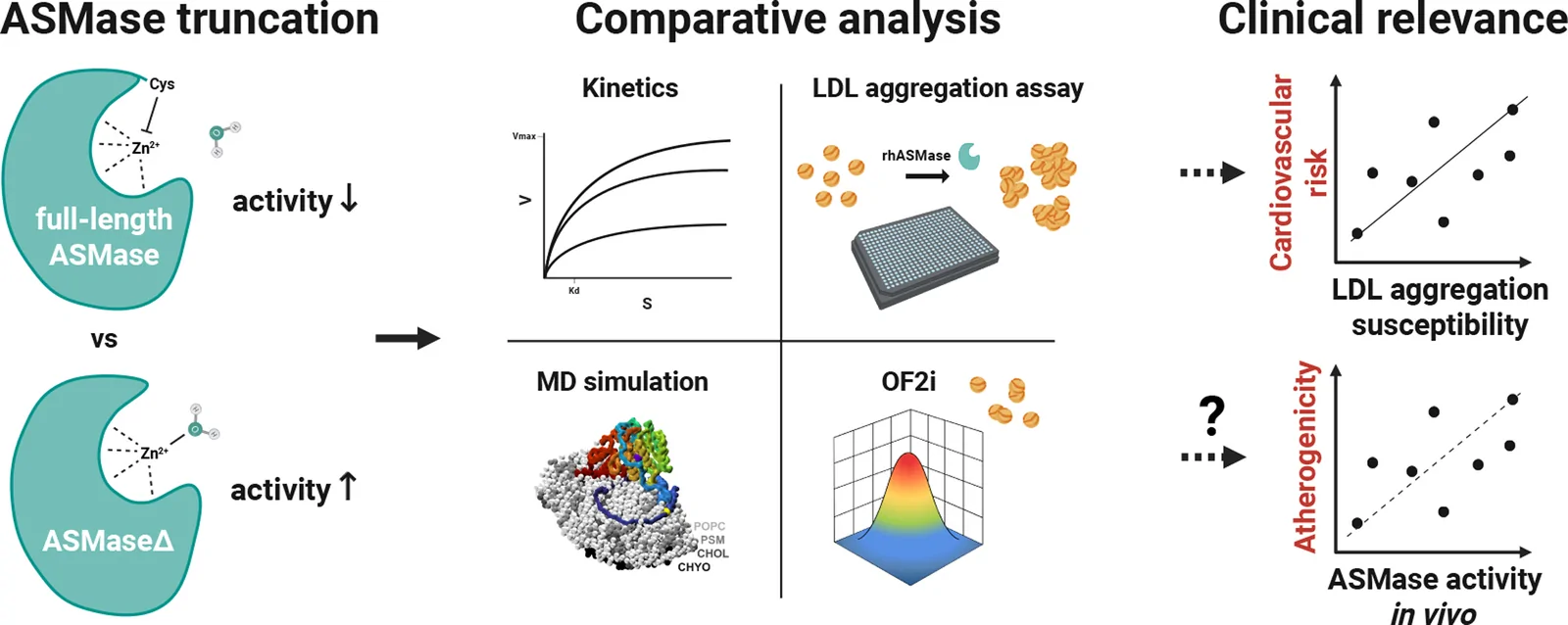

Low-density lipoprotein (LDL) plays a central role in lipid transport and cholesterol homeostasis while also being a key contributor to cardiovascular disease when it undergoes pathogenic modifications in the arterial wall. LDL particles consist of hydrophobic core lipids, amphipathic surface monolayer of lipids, and a single copy of apolipoprotein B-100 (apoB-100) surrounding the particle. Apo-B100 is the major protein component of LDL, maintaining its structural and functional integrity. The particle is a dynamic structure, the properties of which depend on its lipid composition as well as the conformation of apoB-100 (1, 2, 3, 4).

Under physiological conditions, LDL is efficiently cleared from circulation; however, LDL can be retained in the subendothelial space of the arterial wall, the intima, leading to the subsequent formation of an atherosclerotic plaque. In the intima, LDL can undergo various modifications upon exposure to, for example, oxidative, lipolytic, and proteolytic factors and enzymes. These events alter the properties and structural integrity of the LDL particles, leading to their increased retention and aggregation in the arterial wall (5, 6, 7, 8, 9).

While apoB-100 makes up about 20–25% of the LDL particle, the most part represents a complex mixture of lipids, where cholesteryl esters, the transport form of cholesterol, make up 40–50% of the core. The surface phospholipid monolayer comprises 20–25% of LDL, primarily phosphatidylcholine (PC) and sphingomyelin (SM), which maintain membrane rigidity and facilitate interactions. Additionally, triglycerides (5–10%) contribute to LDL density. Minor lipids, such as ceramides and oxidized lipids (1–5%), can accumulate in LDL under pathological conditions and increase atherogenic risk. The lipid composition of LDL directly affects its stability and susceptibility to various modifications (2, 3, 10).

Among LDL lipid components, SM is a key element indicating the particle’s susceptibility to enzymatic modification, which is one of the crucial steps in the development of atherosclerosis. In particular, the enzymatic breakdown of SM by acid sphingomyelinase (ASMase, *SMPD1*) generates ceramide-rich domains, promoting changes in lipid nanoenvironment and apoB-100 conformation in LDL (9, 11, 12). These changes destabilize the LDL particles and cause their aggregation, increased binding to proteoglycans, and enhanced uptake by macrophages, ultimately contributing to atherosclerotic plaque formation (2, 9). Particularly, oxidized LDL has been shown to be more prone to hydrolysis by ASMase and aggregation (13). Exploring the full role of ASMase in atherogenesis is crucial for a broader understanding of the progression of atherosclerosis as a complex process.

ASMase hydrolyzes sphingomyelin into ceramide and phosphorylcholine. Its lysosomal form (L-ASMase) is essential for sphingomyelin metabolism within lysosomes in the acidic environment, while the secretory form (S-ASMase) contributes to extracellular membrane remodeling and signaling at a more neutral pH (14, 15). The enzymatic activity toward SM has been shown to be lower in S-ASMase, compared to L-ASMase, due to the presence of C-terminal cysteine (Cys629), presumably causing weaker coordination of a water molecule with Zn^2+^ ion needed for hydrolysis (15, 16). Extracellular S-ASMase is found in atherosclerotic plaques and is known to play a key role in atherogenesis (11, 17, 18). Indeed, the susceptibility of LDL particles to ASMase-mediated aggregation has been shown to associate with future risk for cardiovascular events (3, 19, 20).

We have previously developed a method to measure LDL aggregation susceptibility in vitro, which was demonstrated to be higher in ASCVD cases (3). The method includes the isolation of LDL particles from patient samples by sequential ultracentrifugation followed by LDL aggregation in vitro, which is induced by recombinant human ASMase (rhASMase). The formation and size of LDL aggregates are followed with dynamic light scattering (DLS) (supplemental Fig. S1). The inflection point of the aggregation curve is then determined, reflecting the LDL aggregation rate and susceptibility through the shape of the curve. The results have indicated an association between aggregation-prone LDL and cardiovascular events and deaths, which potentially represents a novel measurable biomarker of ASCVD risk (3, 19, 20).

In this study, we examined rhASMase-mediated lipolysis and subsequent aggregation of LDL particles by comparing three recombinantly produced rhASMase constructs. We designed two rhASMases, which represent a C-terminally truncated L-ASMase version of the enzyme with either N-terminal (*N-His-SMPD1Δ*) or C-terminal (*SMPD1Δ-His-C*) polyhistidine tag. These enzymes were compared with the N-terminally His-tagged full-length rhASMase (*FL SMPD1*) using both SM and isolated LDL particles as substrates for enzyme activity measurements. To estimate the interactions of rhASMases with a lipid surface and their structural differences, we also performed Molecular Dynamics (MD) simulations. In addition, using optofluidic force induction (OF2i), we assessed the very early steps and dynamics of LDL aggregation during rhASMase-mediated lipolysis.

## Materials and methods

### Expression of recombinant SMPD1 variants

The rhASMase cDNAs were based on homo sapiens *SMPD1* sequence (GenBank: AB209775.1) and were synthesized by Gene Universal. The constructs named *N-His-SMPD1Δ* and *SMPD1Δ-His-C* are 1821 bp long, encoding 580 aa lysosomal *SMPD1*. The cDNA of the full-length ASMase (*FL SMPD1*) has an open reading frame of 1830 bp encoding 583 aa. The plasmid was based on the piggyBac expression vector system containing a CAG promoter and a puromycin selection marker gene (*pac*).

The sequences of *N-His-SMPD1Δ* and *SMPD1Δ-His-C* (606 aa) consisted of 580 aa ASMase Leu47-Pro626 sequence, 18 aa albumin signal peptide, and 8-histidine tag. The full-length ASMase (*FL SMPD1*) sequence (609 aa) consisted of 583 aa (Leu47-Cys629) ASMase sequence, 18 aa albumin signal peptide, and an N-terminal 8-histidine tag.

The rhASMases were produced in stably transfected HEK293F cells in suspension (5 days, +33°C, 5% CO_2_, 120 rpm) in serum-free OptiCHO™ Medium (Gibco™). The proteins were purified by immobilized metal affinity chromatography (IMAC, Protino® Ni-NTA 1 ml, Macherey-Nagel) via polyhistidine tags and dialyzed in 5 mM Tris pH 7.4, 150 mM NaCl, and 0.1 mM ZnCl_2_ for storage. The rhASMase concentrations were measured by light absorption and calculated using the extinction coefficient 142,290 M^-1^ cm^-1^ based on the amino acid sequence (Expasy ProtParam).

### RhASMase activity measurements

SM (Sigma-Aldrich, #85615) hydrolysis by rhASMase was determined by measuring phosphorylcholine from the samples as a reaction product following the two-step Amplex Red Sphingomyelinase Assay protocol (Invitrogen) and using the phosphorylcholine standard curve. The 5 mM SM stock for the reactions was prepared according to the same Amplex Red assay protocol in 2% Triton X-100. The stoichiometry of SM hydrolysis and phosphorylcholine as a product is 1:1. In the standard measurement, rhASMase was incubated for 30 min (if not indicated otherwise) with the substrate at a stated pH, which was followed by a 30 min incubation with Amplex Red working solution in Tris-HCl 100 mM pH 8.

The phosphatidylcholine (PC, Avanti Polar Lipids, #850457P) stock was prepared by the same protocol as SM, as well as the measurement of ASMase reaction product (phosphorylcholine). Ceramide-1-Phosphate (C1P, Avanti Research, #860531P) stock was solubilized in chloroform:methanol (C:M 6:1) and then diluted to 1.5 mM stock with 2% Triton X-100. The C1P stock was additionally solubilized by sonication and solvents were evaporated in the sample tubes before ASMase reaction. The formation of free phosphate as an ASMase reaction product was measured with the Malachite Green Phosphate Assay Kit (Sigma-Aldrich, MAK307). For measurement of BMP hydrolysis by rhASMases, BMP solvent (C:M 9:1) was evaporated by nitrogen before the reaction in sample tubes and then lipids were extracted and cleaned according to the Folch method (21) after the reactions. The extracted lipids were injected into an Agilent 1290 Infinity LC system coupled to an Agilent 6490 Triple Quadrupole MS system. BMP was detected in negative ionization mode, and raw peak intensities were normalized to an external BMP standard and the negative control samples to estimate relative BMP abundance and assess enzyme activity. All incubations were performed at 37°C and the reactions were stopped at an indicated time point by incubation on ice.

Storage and freeze–thaw stability were determined by measuring activity of the purified rhASMase upon storage at −80°C as indicated in Supplemental data (supplemental Fig. S3). The batches of rhASMases, as well as LDL and SM, were kept the same within any repetitive or continuous experiment to exclude the inter-batch effects.

### Kinetics measurements of rhASMases

The kinetic parameters were obtained using the Michaelis-Menten kinetics model. The initial velocity (V_i_) was measured at the various SM and LDL concentrations at pH 5 and 5.5 at 37°C in 20 mM MES, 150 mM NaCl, 2 mM MgCl_2_, 2 mM CaCl_2_ and 0.2 mM ZnCl_2_. The reactions were stopped at certain time points (5, 10, 15, 20 min) by incubation on ice. The concentrations of rhASMase for SM and LDL assays were 0.00133 μM (0.0001 μg/μl) and 0.666 μM (0.05 μg/μl), respectively. The final volume of each reaction was 100 μl.

### Molecular dynamics simulations

Five conformations per rhASMase were generated with ColabFold using PDB 5I81 as a template (22, 23). Their residue charges were set to pH 5.5 and they were coarse-grained with Martinize2 using the Martini3-IDP force field with default GōMartini settings and by defining residues 39 to 87 as an IDR region (24, 25, 26, 27, 28). Each conformation was placed into the water phase in a 21 nm × 21 nm × 28 nm (XYZ) box with a lipid trilayer mimicking LDL surface composition with a 1000 cholesteryl oleate (CHYO) layer surrounded by two phospholipid-cholesterol layers (2). Each phospholipid-cholesterol layer contains 470 palmitoyloleoylphosphatidylcholine (POPC), 180 palmitoylsphingomyelin (PSM), and 350 cholesterol (CHOL) (29, 30, 31, 32, 33). Systems were neutralized either with ∼20 chloride counterions or with 834 NaCl (150 mM), 11 MgCl2 (2 mM), 11 CaCl2 (2 mM) and ∼20 chlorides. This afforded 30 systems in total, which were simulated for 21 μs with a 20 fs timestep using GROMACS software package (34). The short-range neighbor list cut-off was 1.35 nm, van der Waals interactions were handled with a 1.1 nm cut-off and electrostatics with a reaction-field cut-off of 1.1 nm and a relative dielectric constant of 15 and infinity for the reaction field. A V-rescale thermostat was set to 310 K, and a C-rescale semi-isotropic barostat to 1 bar (35, 36). Constraints were handled with LINCS with a highest order of 8 and number of iterations of 2 (37). Description of the system preparation in further detail and analyses are available in Supplemental Methods S1.

### Full-length rhASMase activation by carboxypeptidase Y

*FL SMPD1* was incubated with Carboxypeptidase Y (CPY, Sigma) at the indicated molar concentration ratios in 50 mM MES buffer pH 6.75 for 1 h at room temperature and then subjected to the activity assay with 0.5 mM SM at pH 5.5 (30 min, 37°C) as described in *RhASMase activity measurements* section.

### LDL isolation and aggregation assay

LDL was isolated from blood plasma or serum by D_2_O-based sequential ultracentrifugation protocol (d = 1.019–1.063 g/ml) (20, 38). The concentrations of LDL are expressed as protein concentration measured by the BCA protein assay (Pierce™) using bovine serum albumin as a standard.

The LDL aggregation was induced by incubation with rhASMase in 20 mM MES pH 5.5, 150 mM NaCl, 2 mM MgCl_2_, 2 mM CaCl_2_, and 0.2 mM ZnCl_2_ at 37°C. Standard concentrations for the LDL aggregation assay were 0.1 mg/ml for LDL and 0.025 mg/ml for rhASMase. The formation of LDL aggregates was followed and measured by intensity-based DLS (DynaPro NanoStar, Wyatt Technology) in duplicate. The LDL aggregate size (nm) is expressed as a hydrodynamic radius of the particles and aggregates. The inter-assay coefficient of variation (CV) for the LDL aggregation assay was determined using *N-His-SMPD1Δ* as 10.9% (n = 24) based on one donor’s LDL isolated each time separately.

### LDL-SM content measurement

High-performance thin-layer chromatography (HPTLC) was used to separate and quantify the major polar lipids and neutral lipids using the solvent systems described previously (7). Samples were applied to silica plates using a CAMAG Automatic TLC Sampler 4. Lipid concentrations were determined by scanning charred lipid bands under UV light with a CAMAG TLC Scanner 3. Peak areas were integrated using winCATS software (CAMAG version 1.1.3.0) and compared to lipid standard samples.

### Optofluidic force induction (OF2i)

OF2i was applied to investigate the early steps of rhASMase-induced LDL aggregation. This technology for characterization of nanoparticles relies on the properties of a vortex-shaped laser beam moving the particles in spiral trajectories through a microfluidic capillary, allowing the measurement of each particle separately and resulting in continuous particle size distributions (39). The LDL was isolated by sequential ultracentrifugation (KBr, 1.019–1.063 g/ml), purified with size exclusion chromatography and dialyzed in 140 mM NaCl prior to the measurements. The aggregation of LDL (0.01 mg/ml) was induced by rhASMase variants (0.03 mg/ml) in 20 mM MES pH 5.5, 150 mM NaCl, 2 mM MgCl_2_, 2 mM CaCl_2_ and 0.2 mM ZnCl_2_ at 37°C. The measured values represent concentration and single-particle-based radius of hydration (R_h_) value histograms (40). These histograms represent number-based size distributions over time, which were averaged over 120 s for better representation.

### Biological samples for LDL isolation

Human plasma and serum samples for LDL isolation were collected from healthy volunteers with informed consent according to the Declaration of Helsinki principles. The collection of the samples was approved by the HUS Regional Committee on Medical Research Ethics of the Helsinki University Hospital (Helsinki, Finland) (HUS/2148/2019). Human plasma used for LDL isolation for rhASMase kinetics, storage and F/T stability measurements was obtained as a plasma pool (3 healthy donors) from Finnish Red Cross Blood Service (approval numbers: 43/2023 and 36/2024 obtained for Wihuri Research Institute). LDL isolated from human serum samples from Inner-Savo DM65+ cohort (approval Research Ethics Committee of the Northern Savo Hospital District, Kuopio, Finland, #256/2015) was used for comparison between *N-His-SMPD1Δ* and *SMPD1Δ-His-C* for inducing LDL aggregation as well as the association between LDL aggregation and SM hydrolysis (41).

Optofluidic force induction studies were performed using LDL isolated from human plasma of healthy normolipidemic volunteers. The plasma was obtained from the Department of Blood Group Serology and Transfusion Medicine (Medical University of Graz, Austria), after written informed consent, according to a protocol approved by the Institutional Review Board of the Medical University of Graz.

### Statistical analysis

In measuring rhASMase activity, the reaction velocity was determined as the value of the best-fit slope obtained from a simple linear regression of the concentration of a product over time. The kinetic parameters of rhASMases were obtained using the Michaelis-Menten nonlinear regression model and compared with the Kruskal–Wallis test. In LDL aggregation assays, nonlinear regression analysis was used to fit time-versus-aggregate-size aggregation curves to determine the inflection points as a measure of LDL aggregation susceptibility. The inflection points of LDL aggregation induced by three rhASMases were compared using paired one-way ANOVA, and the differences between the two truncated enzymes were compared using the Wilcoxon paired test. All mentioned analyses and data plotting were performed in GraphPad Prism 11.0.0. The data obtained from OF2i was processed and plotted in MATLAB R2024b.

## Results

### Properties of the recombinant SMPD1 variants

Three purified rhASMases resulted in ∼75 kDa glycosylated protein with a minor fraction of ∼57 kDa (supplemental Fig. S2), presumably resulting from an N-terminal cleavage and other post-translational modifications of the 75 kDa form in accordance with previous research (42). Both N-terminally tagged rhASMases gave weaker signals with anti-hASMase pAb, which is specific to the full-length *SMPD1* sequence. The reduced interaction might be caused by changes in conformation and accessibility of the N-terminus due to the presence of a tag.

The purified enzymes were compared for their biochemical properties and ability to induce LDL aggregation. The pH profile (supplemental Fig. S3A) showed pH optimum peaks of 4.5–5, typical for ASMase. The enzyme stability at −80°C was evaluated by linear regression of the measured activity over a storage period of 0–12 months using SM and 12–23 months using LDL as a substrate (supplemental Fig. S3C). The regression slopes were not significantly different from zero (*P* > 0.05), meaning no significant activity loss except for *FL SMPD1* toward SM, which showed a negative slope significantly deviating from zero (*P* = 0.006, *R*^2^ = 0.99), indicating a loss of −2.6% of activity per month. The truncated rhASMases showed resistance to freeze-thaw (F/T) cycles, whereas the mean activity of *FL SMPD1* significantly decreased after the third F/T cycle to 57% of the baseline activity (*P* = 0.005). (supplemental Fig. S3B).

### Kinetics of rhASMases

The enzyme kinetics of the rhASMases was investigated using the Michaelis-Menten model, providing such kinetic parameters as the maximum velocity (V_max_), the Michaelis constant (K_m_) and turnover number (k_cat_). The initial velocities (V_i_) were measured at different SM and LDL concentrations at pH 5 and 5.5 at 37°C in the presence of Zn^2+^. LDL molar concentration was calculated based on the molecular weight of apolipoprotein B-100 (512 kDa) (one apoB-100 per LDL particle). Accordingly, rhASMase activity toward LDL can be expressed on a per-particle basis. Based on previous reports, an average LDL particle contains approximately 185 SM molecules (2), providing a reference for comparison with rhASMase activity measured using SM as the substrate. The enzyme’s activity toward LDL as a substrate, thus, can be interpreted as rhASMase activity per LDL particle. LDL used for kinetics measurement was isolated from pooled plasma of 3 healthy donors to minimize the impact of interindividual variation in LDL composition on enzyme activity. The differences between the truncated rhASMases were the most prominent at pH 5, while *FL SMPD1* showed the weakest activity in all conditions (Fig. 1). The kinetic values of rhASMases are summarized in Table 1, and specific activity values in supplemental Table S1.

**Fig. 1:**
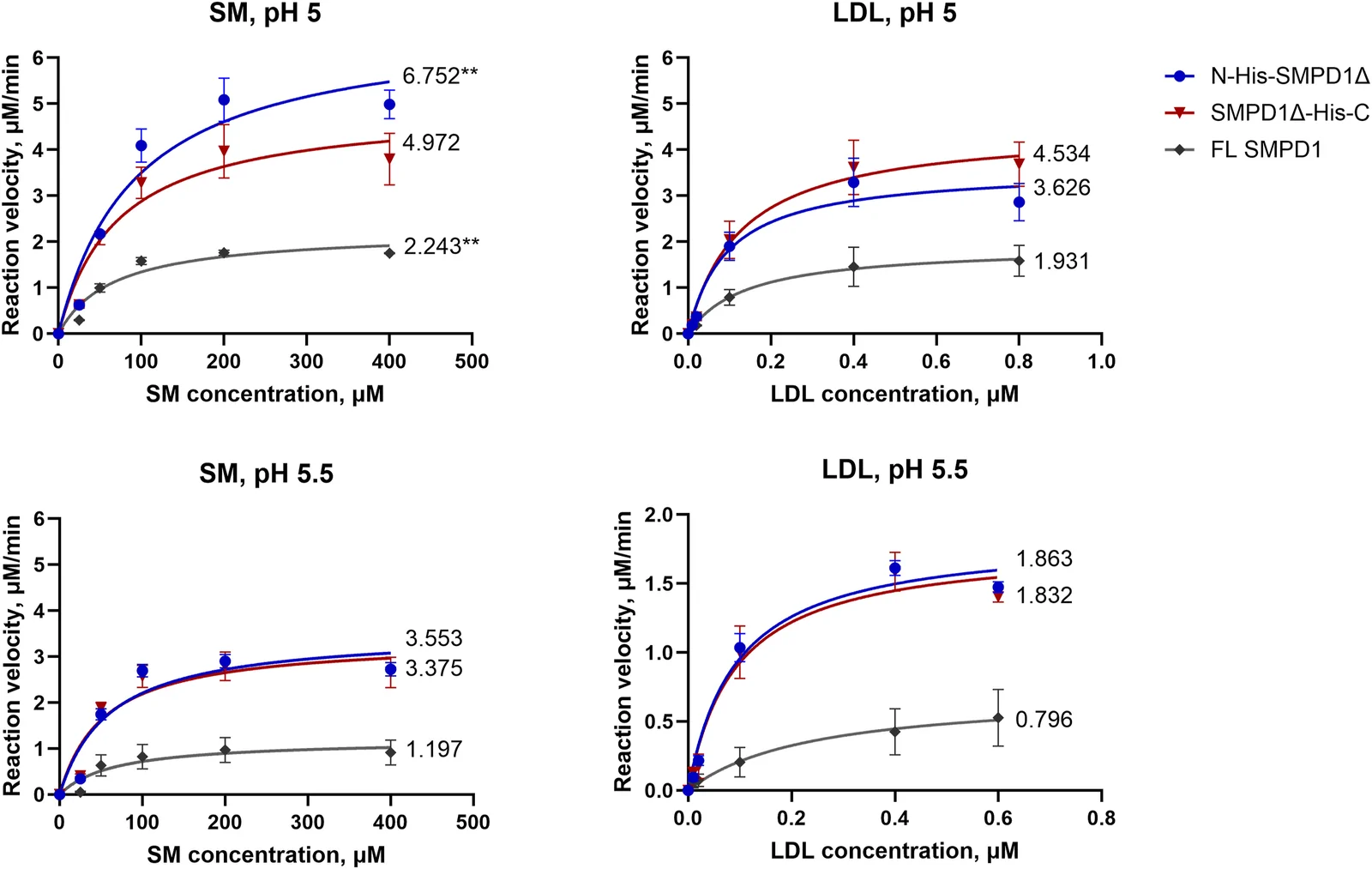
Michaelis-Menten kinetics model for rhASMases with different substrates and conditions. Indicated values represent maximum reaction velocities (V_max_). LDL molar concentration was calculated based on the molecular weight of apoB-100 (512 kDa). 1 mol LDL (apoB-100) on average contains 185 mol SM (2). Experiments were conducted in 3 experimental replicates, and the results are shown as mean ± SEM. ∗∗Kruskal-Wallis test, *P* = 0.0073 (*N-His-SMPD1Δ* vs. *FL SMPD1* SM, pH 5).

**Table 1:** Kinetic parameters of the rhASMases

Conditions	k_cat_, min^−1^		K_m_, μM		V_max_, μM/min		k_cat_/K_m_, μM^−1^ min^−1^	
SM, pH 5								
*N-His-SMPD1Δ*	5077^a^	±415	92.6	±3.3	6.752^a^	±0.552	54.7	±3.2
*SMPD1Δ-His-C*	3738	±618	70.1	±13.2	4.972	±0.822	53.4	±3.2
*FL SMPD1*	1687^a^	±11	67.9	±7.7	2.243^a^	±0.015	25.5	±2.9
LDL, pH 5								
*N-His-SMPD1Δ*	5.443	±0.883	0.099	±0.016	3.626	±0.589	56.5	±9.6
*SMPD1Δ-His-C*	6.807	±0.958	0.136	±0.017	4.534	±0.638	52.3	±10.5
*FL SMPD1*	2.900	±0.798	0.139	±0.035	1.931	±0.532	21.3	±4.8
SM, pH 5.5								
*N-His-SMPD1Δ*	2672	±134	61.1	±4.1	3.553	±0.178	44.0	±2.7
*SMPD1Δ-His-C*	2537	±315	53.4	±4.1	3.375	±0.419	47.1	±2.2
*FL SMPD1*	900	±251	69.1	±5.8	1.197	±0.334	13.8	±5.0
LDL, pH 5.5								
*N-His-SMPD1Δ*	2.798	±0.100	0.098	±0.019	1.863	±0.066	30.2	±4.3
*SMPD1Δ-His-C*	2.750	±0.073	0.110	±0.029	1.832	±0.049	29.9	±9.7
*FL SMPD1*	1.196	±0.280	0.432	±0.200	0.796	±0.187	4.9	±3.0

LDL molar concentration was calculated based on the molecular weight of apolipoprotein B-100 (512 kDa). Experiments were conducted in 3 experimental replicates. The results are reported as mean ± SEM.

a *P* < 0.01. Kruskal-Wallis test, *P* = 0.0073 (*N-His-SMPD1Δ* vs. *FL SMPD1* SM, pH 5).

The kinetics of rhASMases was determined at the optimum pH 5 as well as at pH 5.5, which is the condition of the LDL aggregation assay. Comparing the rhASMases by k_cat_ (=V_max_/[Et]) we found that *N-His-SMPD1Δ* showed the highest k_cat_ with SM at pH 5 – 36% higher than that of *SMPD1Δ-His-C* and 3-fold higher than *FL SMPD1*. However, *SMPD1Δ-His-C* demonstrated the highest activity towards LDL at pH 5 with k_cat_ 25% and 2.3-fold higher than that of *N-His-SMPD1Δ* and *FL SMPD1*, respectively. *FL SMPD1* consistently showed the lowest k_cat_ values, where the most substantial difference was observed with SM at pH 5, resulting in k_cat_ of 1687 min^−1^ in comparison to 5077 min^−1^ of *N-His-SMPD1Δ* (*P* = 0.0073), and at pH 5.5 with k_cat_ of 900 min^−1^ compared to *N-His-SMPD1Δ* k_cat_ of 2672 min^−1^, indicating 3-fold differences.

As for K_m_ values, with LDL as a substrate at pH 5.5, *FL SMPD1* had a 4-fold higher K_m_ (0.432 μM), compared to *N-His-SMPD1Δ* and *SMPD1Δ-His-C* (0.098 μM and 0.110 μM, respectively), suggesting weaker substrate affinity of *FL SMPD1*. The truncated rhASMases showed the opposite affinity patterns at pH 5, where *N-His-SMPD1Δ* had 32% higher K_m_ (92.6 μM) than of *SMPD1Δ-His-C* (70.1 μM) with SM, and 27% lower (0.099 μM) than of *SMPD1Δ-His-C* (0.136 μM) with LDL as a substrate. This suggests somewhat dissimilar affinities to SM and LDL, yet overall K_m_ differences between the truncated variants might not be substantial in terms of the general catalytic activity.

The catalytic efficiency (k_cat_/K_m_), on the other hand, reflects both parameters — the enzyme's catalytic power and substrate affinity. The most substantial difference in k_cat_/K_m_ was observed with LDL at pH 5.5, where *N-His-SMPD1Δ* and *SMPD1Δ-His-C* demonstrated k_cat_/K_m_ of 30.2 μM^−1^ min^−1^ and 29.9 μM^−1^ min^−1^, respectively, indicating 6-fold higher catalytic efficiency compared to *FL SMPD1* (4.9 μM^−1^ min^−1^). Similarly, there was over 3-fold k_cat_/K_m_ difference with SM at pH 5.5. Regardless of individual differences, the overall catalytic efficiency of the truncated rhASMases variants was on a comparable level. However, in most cases, the values for k_cat_/K_m_ and V_max_ of *N-His-SMPD1Δ* were the highest, highlighting its greater enzymatic activity.

For reference, we also compared V_i_ of the rhASMases to the commercial CHO-derived SMPD1 (R&D Systems, 5348-PD-010), which closely corresponds to *SMPD1Δ-His-C* sequence with C-terminal 10-His tag (His62-Pro628). The SM-based reaction velocities of *N-His-SMPD1Δ*, *SMPD1Δ-His-C*, *FL SMPD1* and R&D SMPD1 were determined as 5.267 ± 0.086, 4.757 ± 0.290, 2.041 ± 0.131 and 4.932 ± 0.274 μM/min, respectively (supplemental Fig. S4).

We have also assessed the rhASMase activities with several alternative substrates found in the literature (43, 44, 45). Similar activity patterns were observed with phosphatidylcholine (PC), ceramide-1-phosphate (C1P) and bis(monoacylglycero)phosphate (BMP). The ASMases hydrolyzed PC, a major phospholipid in LDL, only to a limited extent compared with SM, consistent with previous results (supplemental Fig. S5). Across the substrates, *N-His-SMPD1Δ* generally showed the highest activity, whereas *FL SMPD1* showed the lowest with all substrates, except for C1P (supplemental Fig. S5).

### Computational investigation of LDL-rhASMase interactions

To investigate whether the experimentally observed differences between the rhASMase variants are associated with differences in their interactions with the LDL surface, we performed coarse-grained molecular dynamics simulations using an LDL-mimetic lipid system. As the folds of ASMase N- and C-termini have not been determined experimentally, they were predicted with ColabFold using PDB 5I81 as a template (22, 23). We noted that in all of the generated conformations (ten for *FL SMPD1*, five for *N-His-SMPD1Δ* and five for *SMPD1Δ-His-C*) the C-terminus folded over the active site. A representative coarse-grained structure is shown in Figure 2A. Simulations for each rhASMase were conducted with a lipid trilayer mimicking LDL surface composition and with either only neutralizing counterions or with ion concentrations mimicking those used in the in vitro experiments. A trilayer system, used in previous studies on LDL (46, 47) was opted for instead of LDL-sized lipid droplet due to MD simulation capacities. Five replicates, where the ASMase was parametrized with different conformations, were simulated for 21 μs each, bringing the total system count to 30.

**Fig. 2:**
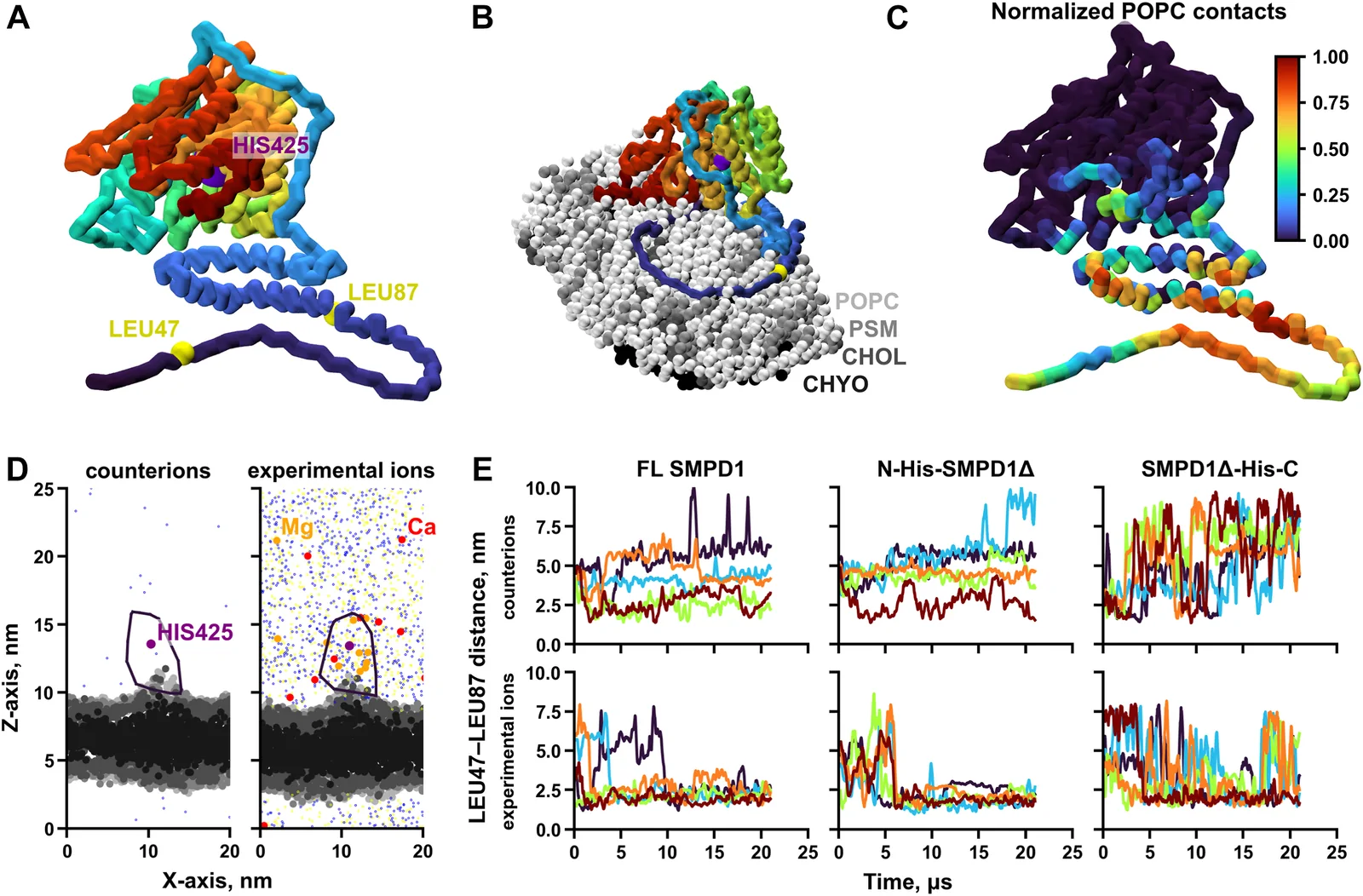
Primary representative results of coarse-grained molecular dynamics simulations. (A) Backbone beads of *FL SMPD1* colored according to residue index (from dark blue of N-terminus to dark red of C-terminus). Additionally, residues LEU47 and LEU87 are colored yellow to mark the disordered N-terminus region, while HIS425 is colored purple to mark a representative residue of the active site. (B) final frame snapshot of *FL SMPD1* bound to LDL surface lipids. (C) *FL SMPD1* residues colored by the number contacts to POPC. Residues in dark red had the highest number of contacts to POPC, while residues in dark blue had almost none. (D) final frame snapshots of *FL SMPD1* counterion and experimental ion systems. Black to gray circles are centers of mass of lipid molecules colored as previously. Small blue and yellow circles are chloride and sodium ion beads, while large orange and red circles are magnesium and calcium ion beads, respectively. The solid dark blue line is the convex hull of *FL SMPD1*, with the purple circle marking the active site residue HIS425. (E) Disordered N-terminus region dynamics of rhASMases for each system.

With only counterions, which balance the electrical charge of the system, ASMases bound to the lipids faster than with experimental ions, and at the end 15 out of 15 were bound, while 6 out of 15 were for experimental ions (supplemental Figs. S6–S8). A representative final frame snapshot showcases the binding mode (Fig. 2B). The kinetics of reaching the final binding mode were investigated further, but no relevant differences were found between rhASMases (supplemental Figs. S9 and S10). Contact analyses show that ASMase primarily utilizes the saposin domain when binding with the active site pointing towards lipids (Fig. 2C and supplemental Figs. S11 and S12). With counterions, the His-tag participates in the interaction, while with experimental ions it does not. Per-residue kinetic analysis did not reveal any relevant differences (supplemental Figs. S13 and S14). The lipids were noted to accommodate rhASMase binding by engaging it with hydrophobic tails and by accessing the active site, leading to a disruption in the trilayer (Fig. 2D and supplemental Figs. S15 and S16).

Overall, the rhASMase variants displayed similar membrane-binding behavior, lipid interactions and membrane-bound orientations in the simulations. The main structural difference observed between the truncated variants was the increased conformational flexibility of the intrinsically disordered N-terminal region in *SMPD1Δ-His-C* compared with *N-His-SMPD1Δ* (Fig. 2E). This is consistent with RMSF analysis of the counterion systems (supplemental Fig. S17). For all rhASMases on average, the N-terminus was more extended in counterion systems compared to experimental ion systems and it is seemingly independent of lipid binding (compare to supplemental Fig. S9). We also noted two phenomena which we believe do not affect our main findings: in 7 counterion systems and in 1 experimental ion system, CHYO molecules clustered into a larger condensed phase (supplemental Figs. S18–S20), and magnesium beads accumulated to ASMase but not calcium beads (supplemental Figs. S21–S22). Extended discussion of these is left to the corresponding supplementary figure captions.

### Proteolytic activation of full-length ASMase

The data from the kinetic experiments suggested that truncation of the rhASMase improves its activity toward SM as well as toward LDL-SM. To test and highlight the role of the truncation, we treated the full-length rhASMase with increasing concentrations of carboxypeptidase Y (CPY), a serine protease that cleaves amino acids sequentially from the C-terminus. The activities of the CPY-treated rhASMases toward SM were then measured. The results showed that the relative activity of *FL SMPD1* was dose-dependently increased by treatment with CPY up to 249% (Fig. 3).

**Fig. 3:**
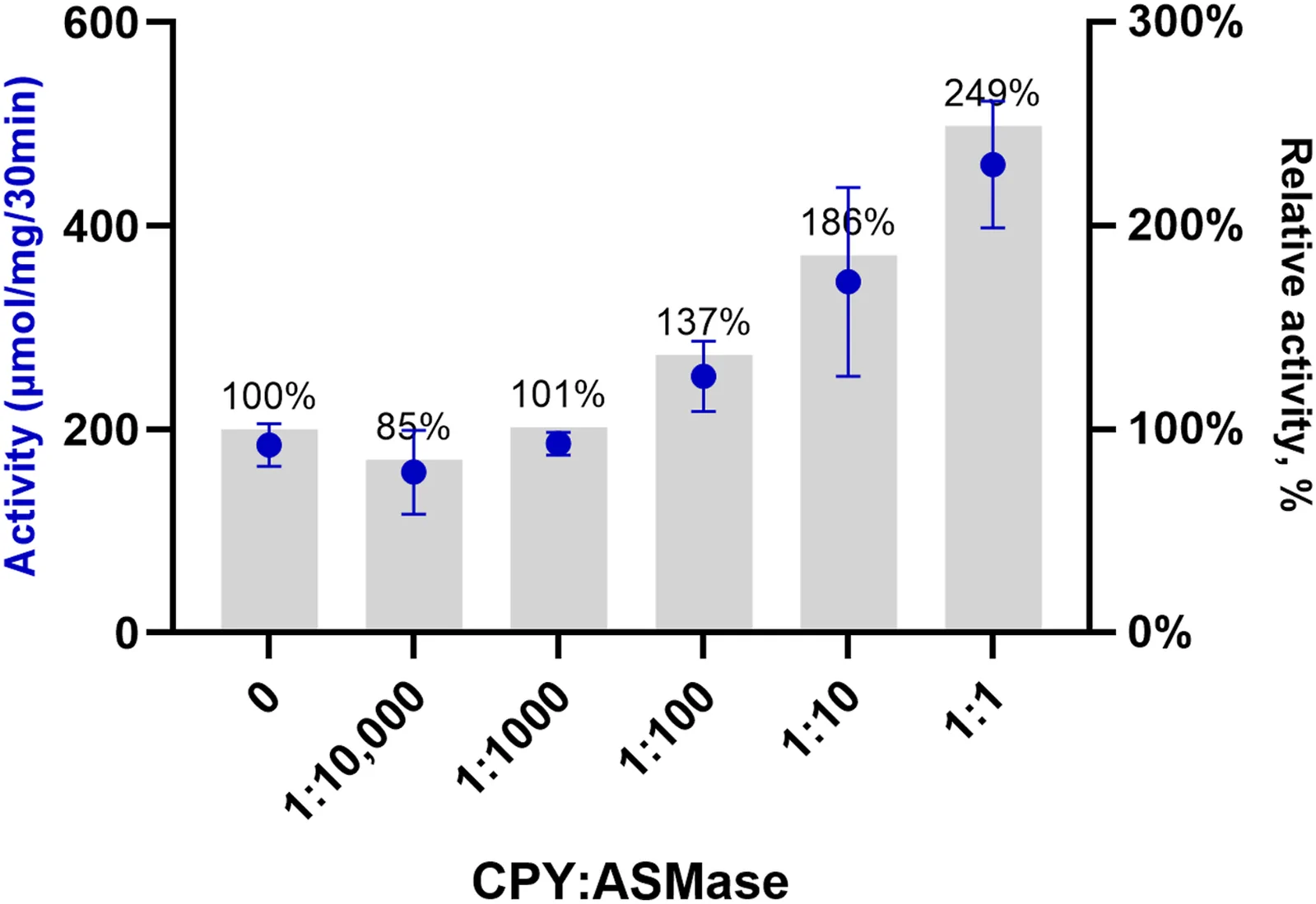
The FL SMPD1 activity increases upon incubation with an increasing CPY:ASMase ratio. The activity is expressed as μmol of the reaction product (substrate: SM, product: phosphorylcholine) per mg of rhASMase per 30 min of incubation. Measurements were conducted in experimental duplicates and represented as mean ± SD. The relative activity, represented in bars, relates to the baseline (ratio 0, 100%), which shows the activity without CPY in the incubation.

### LDL aggregation and SM hydrolysis

We next examined whether the differences in the rhASMase variants translate into differences in LDL aggregation kinetics. For this purpose, we measured rhASMase-induced LDL aggregation using an assay based on measurement of forming LDL aggregates and aggregate sizes using DLS (supplemental Fig. S1). As a measure of LDL aggregation, the assay compares the inflection points (EC50) of the aggregation curves. The less time to reach the inflection point, the faster the aggregation. The assays demonstrated more than two-fold differences in EC50, showing significantly slower aggregation (=higher EC50) induced by *FL SMPD1* (7.106 ± 1.371 h) compared to *N-His-SMPD1Δ* (3.161 ± 0.505 h) and *SMPD1Δ-His-C* (3.543 ± 0.521 h) (Fig. 4A, B).

**Fig. 4:**
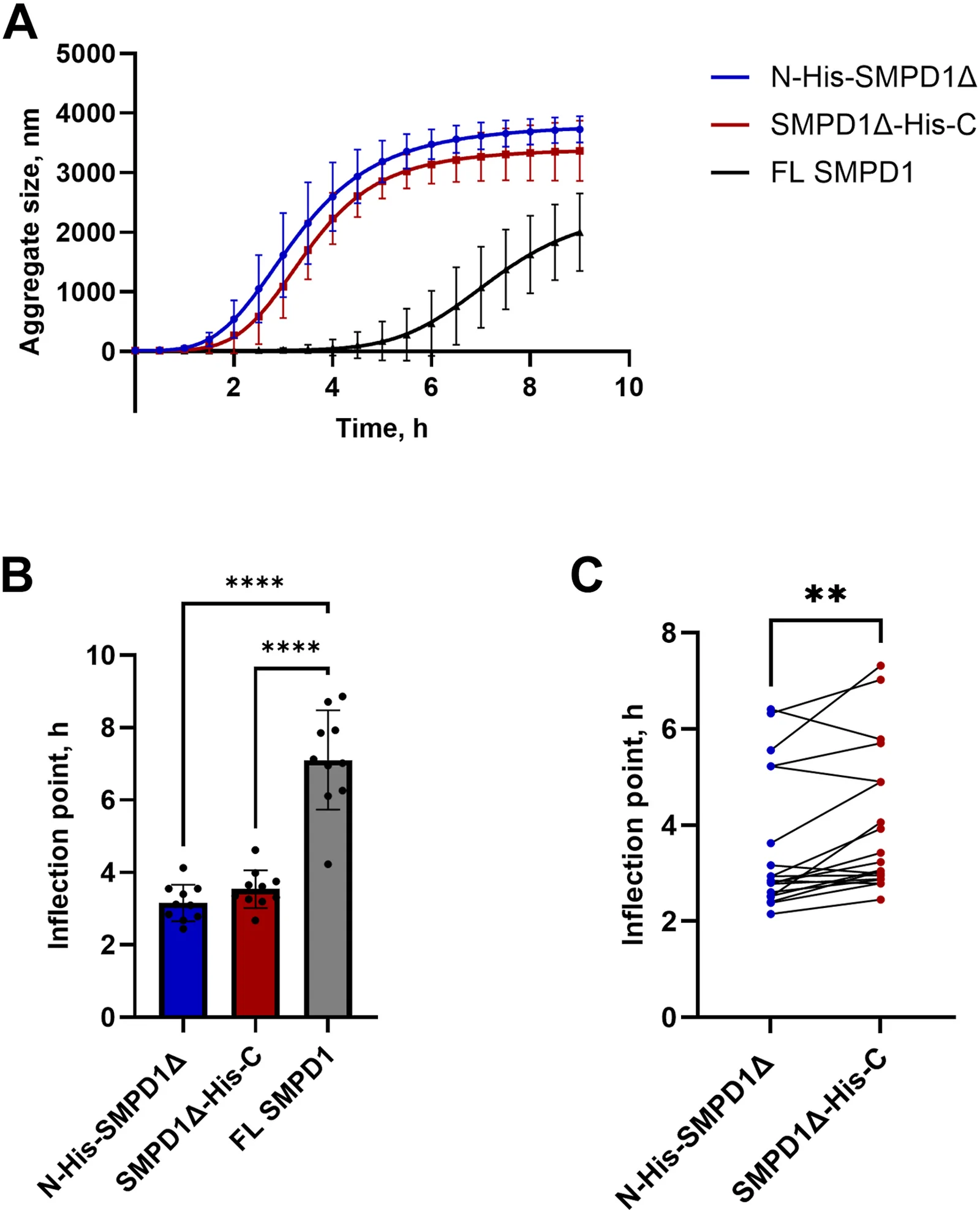
Differences in the LDL aggregation induced by rhASMases variants. (A) Interpolated averaged LDL aggregation curves from various donors (n = 10) induced by the rhASMases and (B) mean inflection point values (±SD) (one-way ANOVA, *P* < 0.0001). (C*)* LDL aggregation differences induced by truncated rhASMases based on the inflection point (EC50). Wilcoxon paired test (*P* = 0.0033, n = 19).

To compare the two truncated variants more precisely, we isolated LDL from 19 donors' (healthy volunteers) plasma samples and examined their aggregation susceptibility. *N-His-SMPD1Δ* induced 12% more rapid LDL aggregation (median EC50 = 2.841 (IQR 2.523–5.220)) than *SMPD1Δ-His-C* (median EC50 = 3.231 (IQR 2.879–4.905)), with a median difference of 0.44 h (≈26 min) (Fig. 4C).

To highlight the role of SM in LDL aggregation, LDL from 16 donors (Inner-Savo cohort) was analyzed with the LDL aggregation assay, along with the formation of the SM hydrolysis product (phosphorylcholine), which was measured as a reaction velocity under the same reaction conditions. The inflection points of the LDL aggregation curves demonstrated a correlation with the rhASMase reaction velocities (r = −0.5769, *P* = 0.0193) (Fig. 5A, B). Additionally, we measured the LDL-SM content, which also demonstrated significant correlation with these parameters (Fig. 5C, D), where the higher LDL-SM was associated with faster LDL aggregation (=lower inflection point; r = −0.6035, *P* = 0.0133) and higher rhASMase reaction velocity (r = 0.6785, *P* = 0.0039). The characteristics of plasma donors are available in supplemental Table S2.

**Fig. 5:**
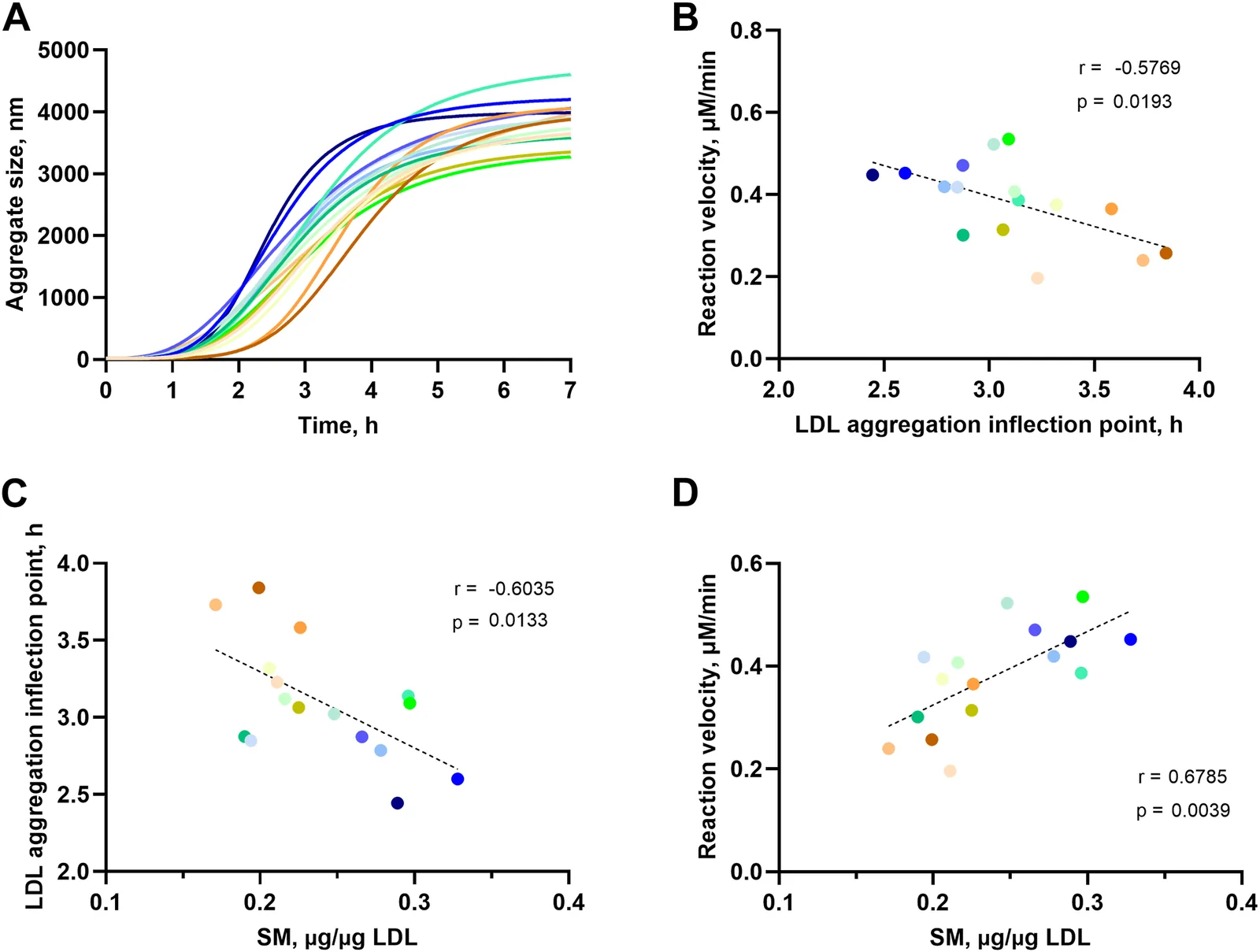
Association between LDL aggregation, LDL-SM and SM hydrolysis (n = 16). (A) LDL aggregation assay curves and (B) the correlation between SM hydrolysis velocity and the inflection point with a linear regression line. (C) The higher LDL-SM content is associated with faster LDL aggregation (=lower inflection point) and (D) higher rhASMase reaction velocity. The assays were conducted using N-*His-SMPD1Δ* in duplicates; plotted data points represent mean values.

### Early kinetics of the ASMase-induced LDL aggregation

To investigate the earliest stages of LDL aggregation, we used optofluidic force induction (OF2i). Formation of LDL aggregates is preceded by structural remodeling of the particle, including rearrangement of the surface lipid monolayer and conformational changes in apoB that ultimately enable particle-particle interactions (9). While DLS-based aggregation assay follows changes in the median hydrodynamic size of the entire LDL population, OF2i measures individual particles and continuously determines their size distribution. This enables detection of the first emerging LDL aggregates before they substantially influence the average particle size measured by DLS. Based on the quality assessment of the measurements, we first determined reliable upper time cut-off thresholds for each measurement and monitored the very initial steps of aggregate formation. During the first 25–40 min after addition of the rhASMases, the first LDL aggregates appeared and gradually increased in size, reaching up to 200–400 nm (R_h_) (Fig. 6). The baseline LDL size was approximately 11 nm (DLS, R_h_). Particle concentration per milliliter represented the amount of objects above 120 nm in the polydisperse system, which describes the first detectable aggregates being formed.

**Fig. 6:**
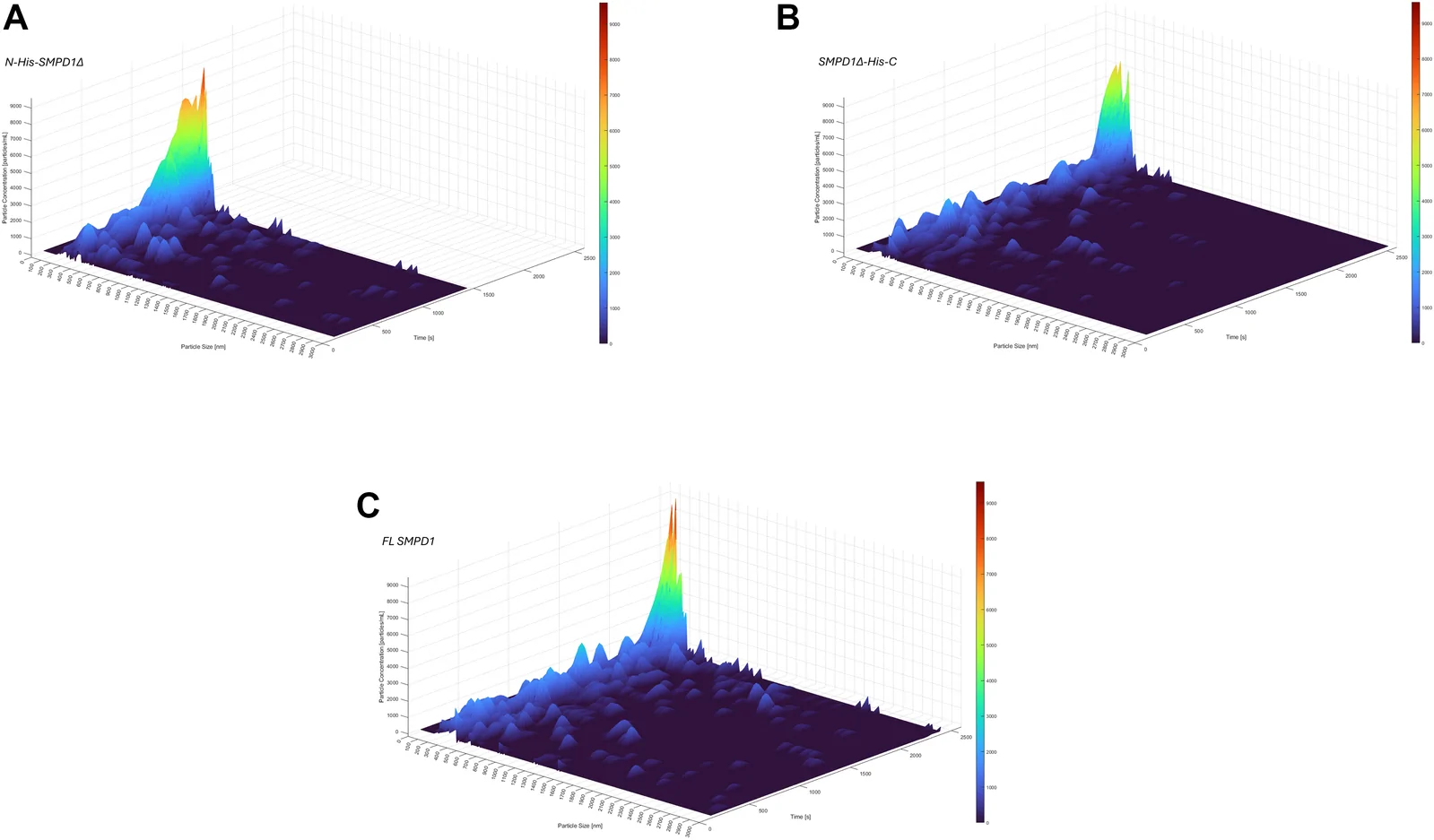
OF2i measurement results of rhASMase-induced LDL aggregation demonstrate particle concentration and size distribution measured over time. Particle concentration per milliliter represents the number of objects above 120 nm. The size (R_h_) distributions were averaged over 120-s intervals for more clear representation. (A) *N-His-SMPD1Δ*; (B) *SMPD1Δ-His-C*; (C) *FL SMPD1*.

Based on the continuous particle size distribution values as a function of time and particle concentration, *N-His-SMPD1Δ* induced the most rapid formation of LDL aggregates during the first 25 min of the measurement (Figs. 6A and 7). Interestingly, *SMPD1Δ-His-C* showed a slower pattern of inducing the LDL aggregation similar to *FL SMPD1* during the early stages of aggregate formation (Figs. 6B, C and 7). *SMPD1Δ-His-C* and *FL SMPD1*-induced reactions reached the similar level of substantial LDL aggregation (particle concentration) as *N-His-SMPD1Δ* only by approx. 40 min after the start of the reaction. The 2D top-view graphs of Figure 6A–C are available in the supplemental materials (supplemental Fig. S23).

**Fig. 7:**
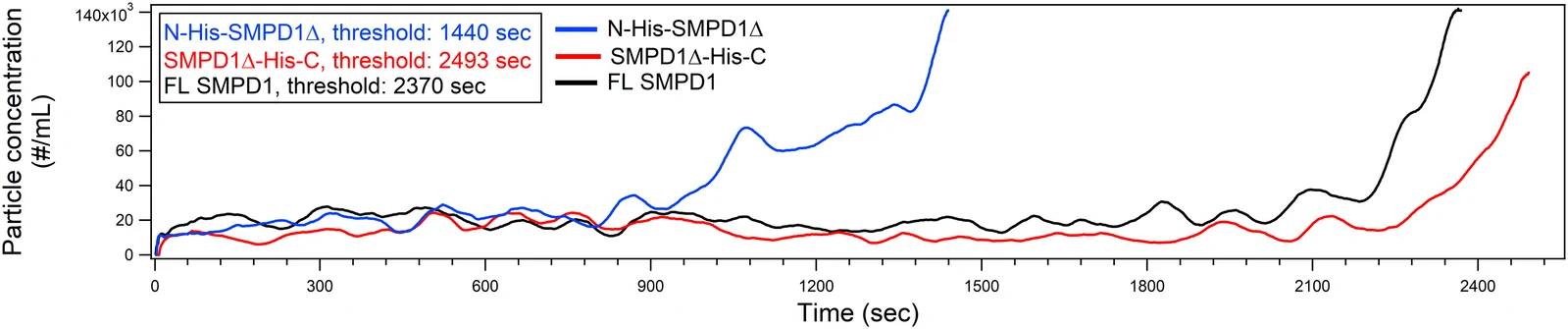
Combined OF2i measurement results of rhASMase-induced LDL aggregation plotted as number-based particle concentration as a function of time. *N-His-SMPD1Δ* induced the fastest early onset of LDL aggregation. Particle concentration per milliliter represents the number of objects above 120 nm (R_h_).

## Discussion

Human ASMase is a key enzyme in sphingolipid metabolism with multiple functions across the body, simultaneously playing one of the key roles in the pathogenesis of atherosclerosis. It is a monomeric glycoprotein containing a saposin domain on the N-terminus, responsible for membrane and substrate binding, and a C-terminal catalytic metallo-phosphatase domain (43). We produced three rhASMase variants for a comparative analysis of their functionality toward LDL particles and in inducing subsequent LDL aggregation: two C-terminally truncated ASMases with His-tags on the opposite termini and a N-terminally tagged full-length version. The truncated rhASMases, representing L-ASMase, displayed 3.5 times higher activity (catalytic efficiency) on average in all conditions as well as two times faster LDL aggregation based on inflection points, compared to *FL SMPD1*. The N-terminally His-tagged truncated *N-His-SMPD1Δ* exhibited the highest activity and expedited LDL aggregation.

The truncation of SMPD1 was previously studied, showing a correlation between the loss of unbound C-terminal cysteine and increased enzyme activity (16). In their study, the CHO-derived rhASMase was analyzed primarily using synthetic SM analogues as well as natural SM as substrates (16). Numerous studies also assessed ASMase activities in various tissues, such as cerebrospinal fluid (CSF) (48) or human fibroblasts (49) using SM analogues. Activity measurements often involve ASMase extracted from the cultured cells (15) or secreted by them and carried out in situ (50) using labelled natural SM or its analogues. Another study involving LDL as a substrate used ceramide-labelled SM incorporated into LDL for measuring ASMase activity secreted by cultured cells (50). Here, we examine the ASMase activity using label-free natural SM as well as the isolated unlabeled human LDL for a better representation of the natural processes.

The kinetics of rhASMase were determined with SM and LDL at both pH 5, which is a general pH optimum for the enzyme, and pH 5.5, which has been suggested to correspond to more acidic conditions of inflamed and hypoxic tissues (51, 52, 53). Both C-terminally truncated constructs showed 3.5-fold higher catalytic efficiency than *FL SMPD1* on average under all four conditions, which is consistent with the previous findings (16). The rhASMases also showed similar activity patterns with alternative ASMase substrates (PC, C1P, BMP).

C-terminal truncation of the full length ASMase reflects the maturation of L-ASMase, involving its truncation on the lysosomal path (54). The loss of unbound C-terminal Cys629 has been suggested to favor the polarization of a water molecule by Zn^2+^ promoting its nucleophilic attack on sphingomyelin’s phosphodiester bond at the catalytic site (16, 22, 54). Indeed, treatment of the full-length ASMase with CPY led up to 2.5-fold increase in its activity, which is assumed to be caused by the loss of cysteine. The results were consistent with those of Qiu *et al.* (2003), where the full-length ASMase activity extensively increased after the incubation with CPY, while also showing the truncation with fluorescent label specific to C-terminal cysteine (16).

The overall catalytic efficiency (k_cat_/K_m_) of the two truncated rhASMase variants can be considered comparable and substantially higher than that of the full-length enzyme. However, the activities of the truncated rhASMases show somewhat different patterns toward SM and LDL as substrates. In comparison, at the optimum pH 5, *N-His-SMPD1Δ* demonstrated 36% higher k_cat_ with SM, while *SMPD1Δ-His-C* showed 25% higher k_cat_ toward LDL. These differences suggest that the His-tag placement may differentially influence the interaction of ASMase with different substrates. The lipid monolayer of LDL particles is a dynamic structure, and to be able to lipolyze LDL-SM, ASMase needs to interact with the lipids on the surface of the particle. The binding of the enzyme to the membrane containing its substrate is facilitated by the N-terminal saposin domain bringing ASMase into close contact with the lipids (55).

To investigate whether the observed differences in the activities of the ASMase variants originate from altered membrane interactions, we performed coarse-grained MD simulations on LDL-mimetic lipid surfaces. All three rhASMase variants displayed a similar mode of interactions, binding the membrane primarily through the saposin domain while orienting the catalytic domain tunnel opening toward the phospholipid layer, consistent with the model proposed by Xiong *et al.* (55). Minimum-distance and contact analyses did not reveal substantial differences in interactions with POPC molecules between the variants. Likewise, membrane-binding kinetics and the orientational distribution of the catalytic domain following membrane association were highly similar, suggesting that neither lipid recognition nor membrane binding can explain the experimentally observed differences in catalytic activities. The most notable difference between the three rhASMases identified in the simulations was the increased flexibility of the N-terminal region in *SMPD1Δ-His-C* relative to the other two variants. Although this increased flexibility did not affect membrane associations in the simulations, it suggests that His-tag placement can induce subtle perturbations in the conformational ensemble of the intrinsically disordered N-terminus of the enzyme. Because intrinsically disordered regions exist as highly dynamic ensembles rather than adopting a single well-defined structure, slight shifts in their conformational landscape may alter transiently their interactions with the catalytic tunnel opening or saposin domain in a stochastic manner, thereby affecting lipid binding and enzymatic activity. However, such effects are challenging to resolve and validate using MD simulations alone (56).

Taken together, our biochemical, enzymatic, and MD simulation data indicate that the markedly enhanced catalytic activity of the two C-terminally truncated rhASMase variants is primarily attributable to removal of the C-terminal region, particularly the loss of Cys629, consistent with previous biochemical and structural studies (15, 16). The increase in activity following CPY treatment of the full-length enzyme further supports this conclusion. In contrast, the more subtle substrate-dependent differences observed between the two truncated variants cannot be explained by altered membrane recognition or binding, as the MD simulations revealed highly similar lipid membrane interactions for all three enzyme variants. Instead, the increased conformational flexibility of the tag-free N-terminus observed in *SMPD1Δ-His-C* suggests that His-tag placement may subtly influence the conformational dynamics of the enzyme. Although the functional significance of this finding remains uncertain, it provides a plausible structural basis for the modest differences observed between the truncated variants.

The LDL aggregation assay was conducted at pH 5.5, where the kinetic differences between the two truncated rhASMases were negligible. However, *N-His-SMPD1Δ* was shown to induce 12% faster LDL aggregation than *SMPD1Δ-His-C* in the assay with a median time difference of ≈26 min. As LDL aggregation correlated with SM hydrolysis velocity, a faster aggregation suggested that the catalytic domain free of the tag is more crucial for enhancing the aggregation reaction. Further measurements supported that LDL aggregation correlates with LDL-SM content and SM hydrolysis velocity, once more highlighting a link between ASMase catalytic activity, LDL composition and LDL aggregation susceptibility. Although the LDL aggregation assay represents a simplified mechanistic model of LDL modification and does not incorporate extracellular matrix components, proteases, or cellular interactions present in the atherosclerotic lesions, it reproduces a key event in atherogenesis and has been shown to associate with cardiovascular risks. Previous studies support positive correlations between LDL-SM content, LDL aggregation susceptibility and cardiovascular events (3, 5, 20, 57, 58, 59).

Importantly, the findings of the present study also suggest that the proteolytic maturation of ASMase may further enhance this process. The markedly increased activity of the truncated enzyme variants indicates that proteolytic removal of the C-terminal region can substantially increase the capacity of ASMase to hydrolyze sphingomyelin within LDL and promote LDL aggregation. Thus, full-length S-ASMase, present in atherosclerotic plaques and expressed by several arterial wall cell types as well as macrophages, can be potentially activated by certain locally secreted proteases (17, 18, 60, 61). This cleavage could substantially increase the activity of a full-length S-ASMase, promoting faster LDL aggregation and accelerated development of atherosclerosis. This aspect of atherogenesis has not been studied previously, leaving a perspective for further exploration of ASMase contribution to the progression of the disease.

All rhASMases exhibited the highest activity at acidic pH 4.5–5 and its complete decrease already at pH 6, which is typical for the enzyme (62). The data available on S-ASMase activity at neutral pH can be rather controversial, considering ASMase functions in vivo and its pH optimum. In particular, S-ASMase in saliva and tear fluid shows the highest activity at pH 4–5 and insignificant activity at pH 7 (63). A similar pH profile was also observed in S-ASMase found in human cerebrospinal fluid, where S-ASMase activity was the highest at pH 5.0–5.6 and less than 10% of the maximum value at pH > 6.4 (48). Certain recombinant ASMases as well as fetal bovine serum S-ASMase have also demonstrated up to 20% of maximum activity at neutral pH (64, 65). The reason might lie in the localization and regulatory mechanisms of ASMase activity, restricting it from excessive hydrolysis in certain tissues.

An established role of S-ASMase in atherogenesis implies that LDL-SM is hydrolyzed in the extracellular space, particularly in the intima, where the environment is normally neutral. Native LDL is not an optimal substrate for ASMase; however, the ability of extracellular ASMase to hydrolyze SM and cause LDL aggregation in vitro as well as LDL retention in vivo under near-neutral intimal conditions has been previously shown (11, 66). Moreover, ASMase has been shown to hydrolyze atherogenic oxidized and proteolyzed LDL as well as SM-rich LDL more efficiently than unmodified LDL, even at neutral pH (13, 67). During the development of an atherosclerotic plaque, local pH can become more acidic due to the ongoing inflammation and, along with elevated Zn^2+^ levels, can contribute to an enhanced activity of S-ASMase and accelerated LDL aggregation (53, 68, 69, 70, 71).

In the LDL aggregation assay, SM hydrolysis starts immediately after the exposure of LDL to rhASMase. However, the formation of the first aggregates approximately during the first 30 min cannot be precisely detected and analyzed using DLS. The OF2i technique allows to gain precise nano-scale particle size distributions of a polydisperse system continuously as a function of time, which makes it a valuable tool for monitoring dynamic processes such as the early stages of rhASMase-induced LDL aggregation. This allowed us to investigate initial LDL aggregate formation patterns, giving clear insights into their size distribution in the 200–400 nm range that precedes the formation of larger aggregates (1–4 μm). The results revealed that *N-His-SMPD1Δ* induced the fastest formation of the first substantial aggregates, which corresponds to the enzyme’s overall activity and LDL aggregation results discussed earlier.

However, considering the comparable overall catalytic efficiency of both truncated rhASMases, this finding can imply that LDL aggregation does not solely depend on total SM hydrolysis velocity. Initial LDL aggregation only occurs after a number of individual particles reach a sufficient surface ceramide accumulation threshold, initiating the critical structural changes in LDL (5, 9, 12). We suggest that *N-His-SMPD1Δ* reaches this state more quickly due to its potentially distinct LDL binding dynamics and a tag-free catalytic domain, which promotes more concentrated lipolysis rather than being distributed evenly across multiple particles.

In conclusion, we found that truncated rhASMases demonstrated 3.5-fold higher catalytic efficiency on average compared to the full-length *FL SMPD1*. *N-His-SMPD1Δ* exhibited the highest catalytic activity and induced the most rapid LDL aggregation. The results highlighted the effect of the C-terminal cysteine loss and subsequent increase in enzymatic activity. The His-tag position also demonstrated the benefit of a tag-free catalytic domain. MD simulations revealed a more dynamic nature of a tag-free saposin domain, although they did not show notable differences in lipid layer docking simulations. Using a novel nanoparticle characterization approach (OF2i), we showed substantial differences in the initiation of LDL aggregation in vitro, where *N-His-SMPD1Δ* induced the fastest aggregation. Truncated rhASMases demonstrated high enzyme activity, storage stability, resistance to freeze-thaw cycles as well as general satisfactory reproducibility, which indicates their suitability for long-term storage and use. We showed a noticeable correlation between LDL aggregation, LDL-SM content and SM hydrolysis rate, highlighting the link between ASMase activity, LDL composition and development of atherosclerosis. A possible activation of a full-length S-ASMase through proteolytic truncation in the arterial wall and its contribution to atherogenesis remains a noteworthy perspective for further research.

## Data availability

Data is available upon request.

## Supplemental data

This article contains supplemental data (72, 73).

## Conflict of interest

Katariina Öörni is an inventor in a patent on the analysis of LDL aggregation susceptibility using rhASMase. All other authors declared no conflict of interest.
