# Supplementary material for: Truncated acid sphingomyelinase induces substantially accelerated LDL aggregation in vitro
Source: J Lipid Res. 2026 Sep 15;67(11):101149. doi: 10.1016/j.jlr.2026.101149 (PMC13770655; doi:10.1016/j.jlr.2026.101149)
Supplement: Supplementary file 1 [file mmc1.pdf]

SUPPLEMENTAL DATA

Truncated acid sphingomyelinase induces substantially accelerated LDL aggregation in vitro

Alina Iakubovskaia[1,2,3], Akseli Niemelä[4], Andrea Benković[5], Andrew House[6], Arja Pasternack[1], Pekka Mäntyselkä[7], Karin Kornmueller[5], Artturi Koivuniemi[4], Olli Ritvos[1], Katariina Öörni[2,3,6]

[1] Department of Physiology, Faculty of Medicine, University of Helsinki, Helsinki, Finland

[2] Molecular and Integrative Biological Sciences, Faculty of Biological and Environmental Sciences, University of Helsinki, Helsinki, Finland

[3] Atherosclerosis Research Laboratory, Wihuri Research Institute, Helsinki, Finland

[4] Division of Pharmaceutical Biosciences, Faculty of Pharmacy, University of Helsinki, Helsinki, Finland

[5] Gottfried Schatz Research Center for Cell Signaling, Metabolism and Aging, Medical Physics and Biophysics Division, Medical University of Graz, Graz, Austria

[6] Helsinki University Lipidomics Unit (HiLIPID), Helsinki Institute of Life Science (HiLIFE), Biocenter Finland, University of Helsinki, Helsinki, Finland

[7] Institute of Public Health and Clinical Nutrition, Faculty of Health Sciences, University of Eastern Finland, Kuopio, Finland, and Teaching Clinic, Wellbeing Services County of North Savo, Kuopio, Finland

Supplemental Table S1. Specific activity of rhASMases based on the mean V_{max} obtained from the kinetic experiments.

	**Specific activity**, μmol/mg/min
**SM, pH 5**	
N-His-SMPD1Δ	67.5 ± 5.5 *
SMPD1Δ-His-C	49.7 ± 8.2
FL SMPD1	22.4 ± 0.2 *
**LDL, pH 5**	
N-His-SMPD1Δ	0.073 ± 0.012
SMPD1Δ-His-C	0.090 ± 0.013
FL SMPD1	0.039 ± 0.010
**SM, pH 5.5**	
N-His-SMPD1Δ	35.5 ± 1.8
SMPD1Δ-His-C	33.8 ± 4.2
FL SMPD1	12.0 ± 3.3
**LDL, pH 5.5**	
N-His-SMPD1Δ	0.037 ± 0.001
SMPD1Δ-His-C	0.037 ± 0.001
FL SMPD1	0.016 ± 0.003

The values are expressed as μmol of the reaction product (phosphorylcholine) per mg of rhASMase per minute. LDL molar concentration was calculated based on the molecular weight of apolipoprotein B-100 (512 kDa). The reactions were incubated at 37°C in 20 mM MES, 150 mM NaCl, 2 mM $MgCl_2$, 2 mM $CaCl_2$ and 0.2 mM $ZnCl_2$. The concentrations of rhASMase for SM and LDL assays were 0.00133 μM (0.0001 μg/μl) and 0.666 μM (0.05 μg/μl), respectively. The measurements were conducted in 3

experimental replicates. The results are reported as mean ± SEM. **N-His-SMPD1Δ* and *FL SMPD1* statistical difference: Kruskal-Wallis test (p=0.0073)

Supplemental Table S2. Inner-Savo cohort donor characteristics (mean±SD, n=16).

**Sex**	males = 11; females = 5
**Age**	77.25±5.18 years
**BMI**	30.82±6.53 kg/m2
**LDL-Cholesterol***	2.59±0.70 mmol/l
**Total cholesterol***	4.49±0.75 mmol/l
**Triglycerides***	1.40±0.65 mmol/l

*Fasting plasma values

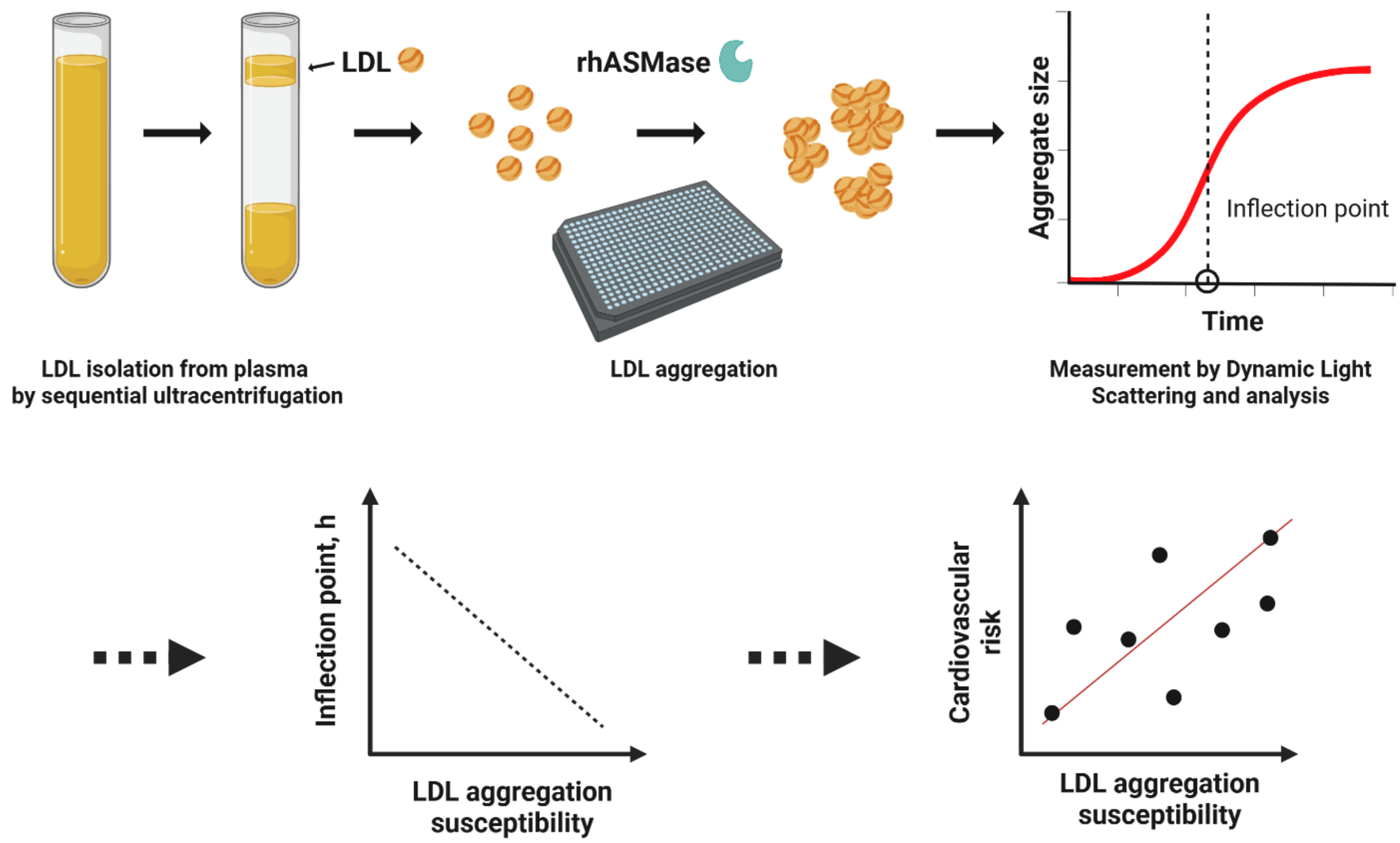

Supplemental Figure S1. The principle of LDL aggregation assay and its biological meaning. The method includes the isolation of LDL particles from patient samples by sequential ultracentrifugation followed by LDL aggregation in vitro, which is induced by recombinant human ASMase (rhASMase) in acidic environment (pH 5.5). The formation and size of LDL aggregates are followed with dynamic light scattering. The inflection point of the aggregation curve is then determined, reflecting the LDL aggregation susceptibility through the shape of the curve. The studies have shown an association between aggregation-prone LDL and cardiovascular events and deaths, which potentially represents a novel measurable biomarker of ASCVD risk.

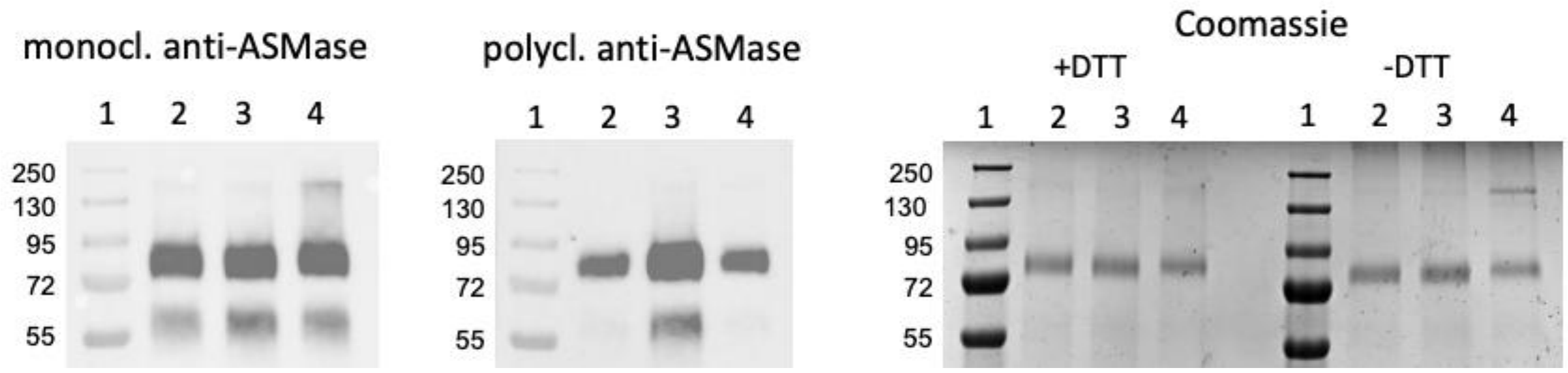

Supplemental Figure S2. Western blot and Coomassie staining profile of rhSMases. 1. MW (kDa) 2. *N-His-SMPD1Δ* 3. *SMPD1Δ-His-C* 4. *FL SMPD1*. Anti-hASMase mAb (OriGene, TA803659) 1:2000; anti-hASMase pAb (Invitrogen, PA5-77047) 1:2000. Load: 1 μg/well.

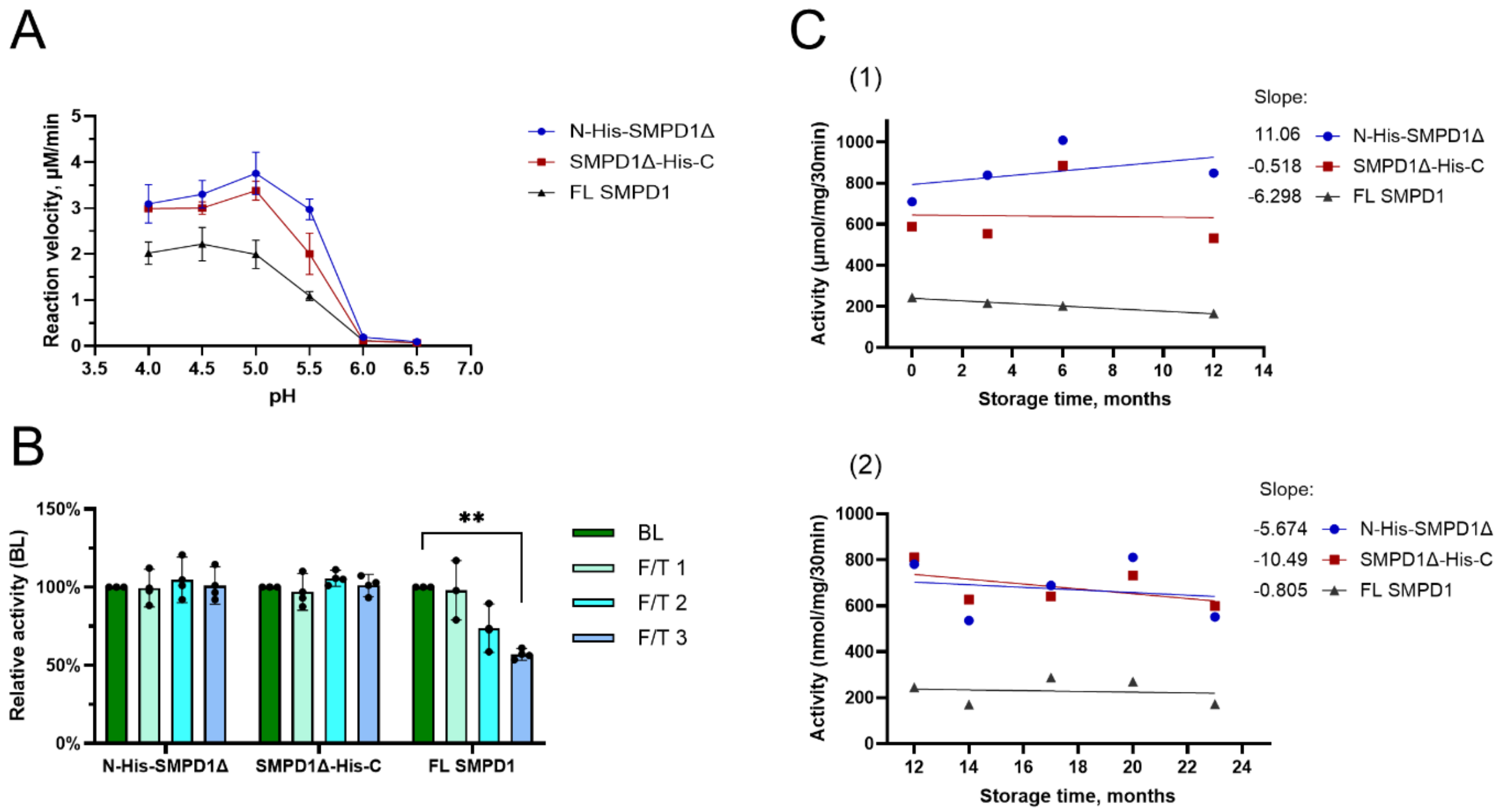

Supplemental Figure S3. Properties of the rhASMase variants. All reactions were incubated at 37°C in 20 mM MES, 150 mM NaCl, 2 mM $MgCl_2$, 2 mM $CaCl_2$ and 0.2 mM $ZnCl_2$. Reaction product (phosphorylcholine) was measured with two-step Amplex Red Sphingomyelinase Assay (Invitrogen). (A) pH profile. Results are represented as mean ±SD, conducted in 3 experimental replicates; (B) Freeze-thaw (F/T) stability of the rhASMase variants. The activity of *FL SMPD1* showed a significant mean decrease by 43% after third F/T cycle (two-way ANOVA, p=0.005). Results are represented as mean ±SD, conducted in 3 experimental replicates (BL=baseline, 0.1 mM SM, 0.1 ng/µl rhASMase, pH 5, 30 min); (C) Linear regression of the rhASMase activity during storage (-80°C). SM (1) and LDL (2) concentrations are 0.5 mM and 0.4 mg/ml, respectively. LDL molar concentration was calculated based on the molecular weight of apolipoprotein B-100 (512 kDa). RhASMase concentrations are 0.1 ng/µl and 0.05 µg/µl for SM and LDL, respectively (pH 5.5, 30 min). Each data point is a mean value of technical duplicates.

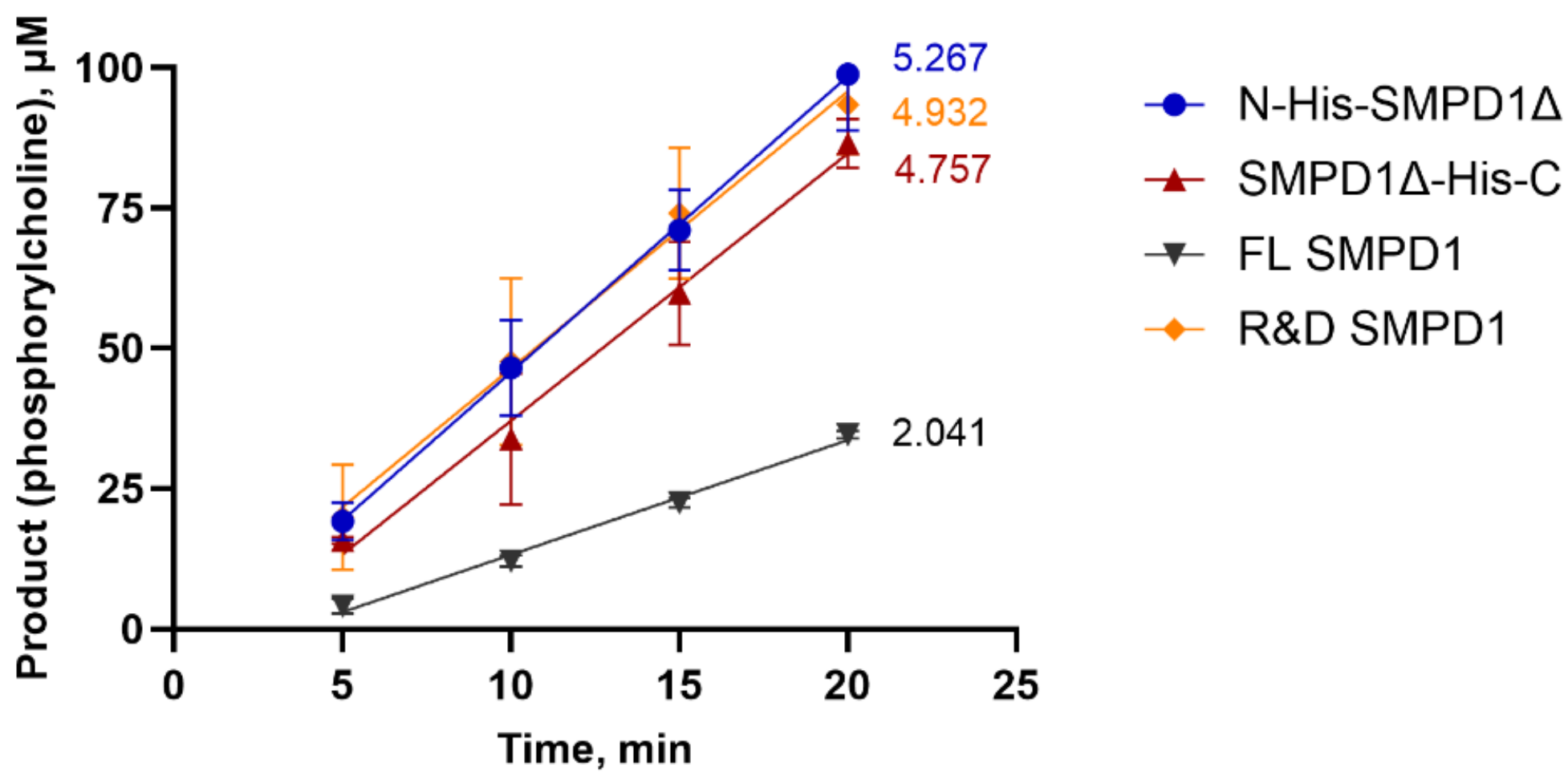

Supplemental Figure S4. Reaction velocities of the in-house produced rhASMases and commercial analogue (R&D Systems, 5348-PD-010). The reactions were incubated at 37°C in 20 mM MES pH 5, 150 mM NaCl, 2 mM $MgCl_2$, 2 mM $CaCl_2$ and 0.2 mM $ZnCl_2$. Concentrations are 0.4 mM and 0.1 ng/µl for SM and rhASMase, respectively. Reaction product (phosphorylcholine) was measured with two-step Amplex Red Sphingomyelinase Assay (Invitrogen). Indicated values represent reaction velocities (µM/min). Experiments were conducted in 3 experimental replicates, and the data is plotted as mean ±SD.

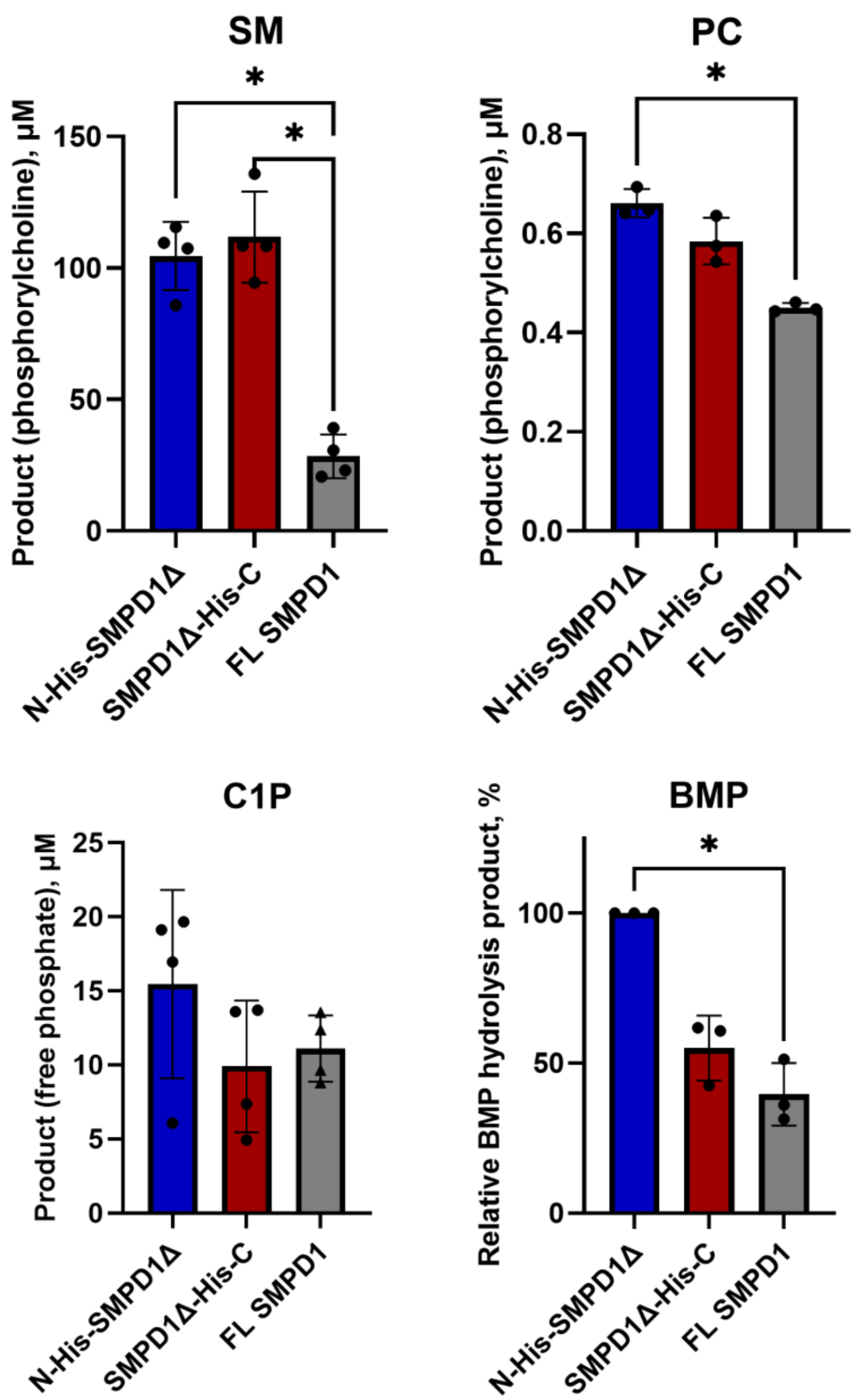

Supplemental Figure S5. RhASMase reaction product formation with SM and alternative substrates: phosphatidylcholine (PC), ceramide-1-phosphate (C1P) and bis(monoacylglycero)phosphate (BMP). *N-His-SMPD1Δ* and *SMPD1Δ-His-C* demonstrated significantly higher activity than *FL SMPD1* with SM (Kruskal-Wallis test: p=0.0241 and p=0.0142, respectively). *N-His-SMPD1Δ* also showed significantly higher activity with PC compared to FL SMPD1 (Kruskal-Wallis test, p=0.0073) as well as with BMP (Kruskal-Wallis test, p=0.01). Data points represent single experiment with mean from technical duplicates

(plotted as mean±SD). All reactions were incubated for 30 min at 37°C in 20 mM MES pH 5, 150 mM NaCl, 2 mM $MgCl_2$, 2 mM $CaCl_2$ and 0.2 mM $ZnCl_2$. Concentrations were 0.5 mM for all substrates and 0.1 ng/μl for rhASMase. SM and PC reaction product was measured with the two-step Amplex Red Sphingomyelinase Assay protocol (Invitrogen), C1P reaction product with Malachite Green Phosphate Assay Kit (Sigma-Aldrich, MAK307) and BMP hydrolysis by measuring the loss of BMP in the sample by LC/MS (represented as a relative hydrolysis product).

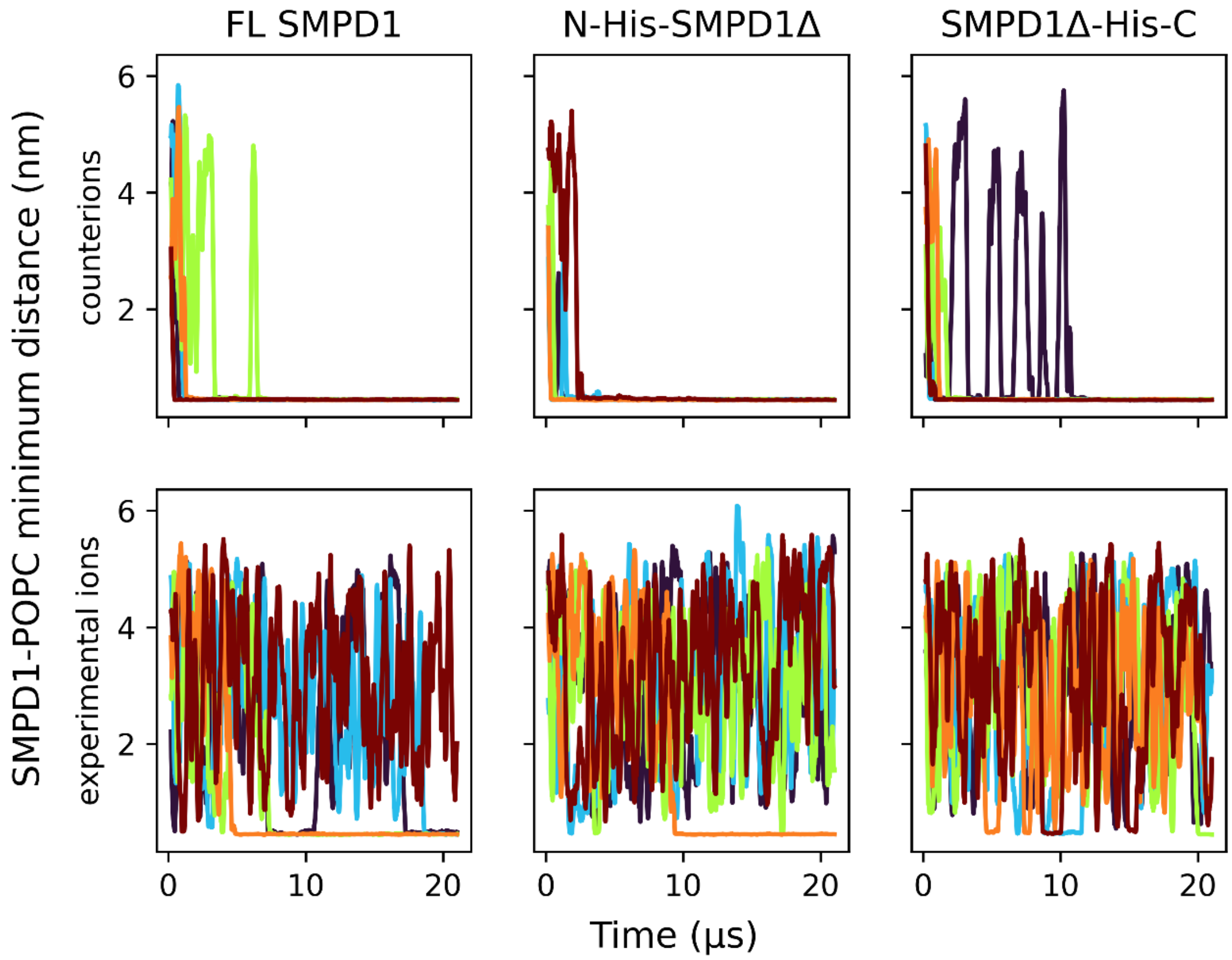

Supplemental Figure S6. Minimum distance between ASMase and POPC as a function of time. Colors represent the replicates (n=5).

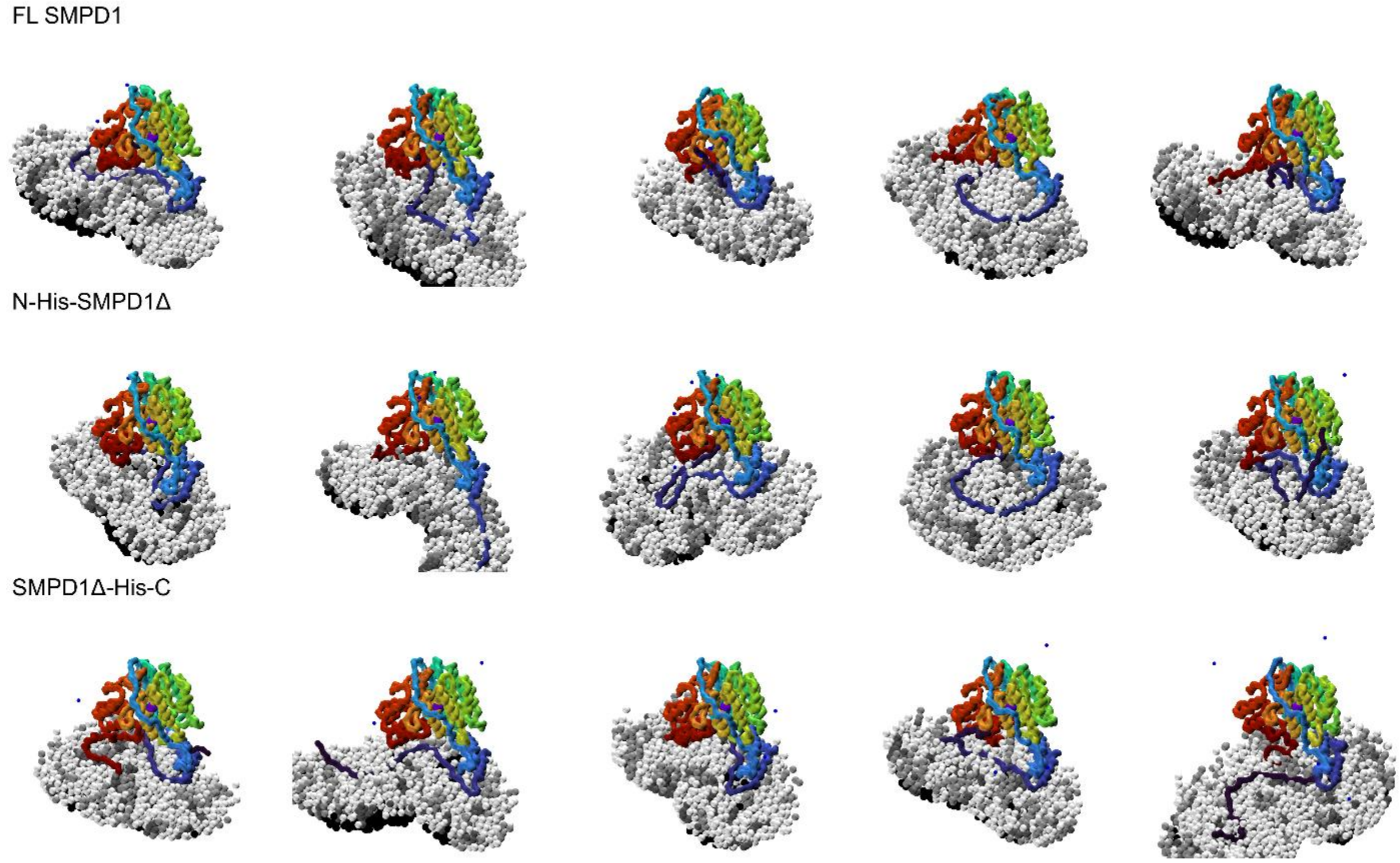

Supplemental Figure S7. Final frame snapshots of counterion systems. Images from left to right represent replicates.

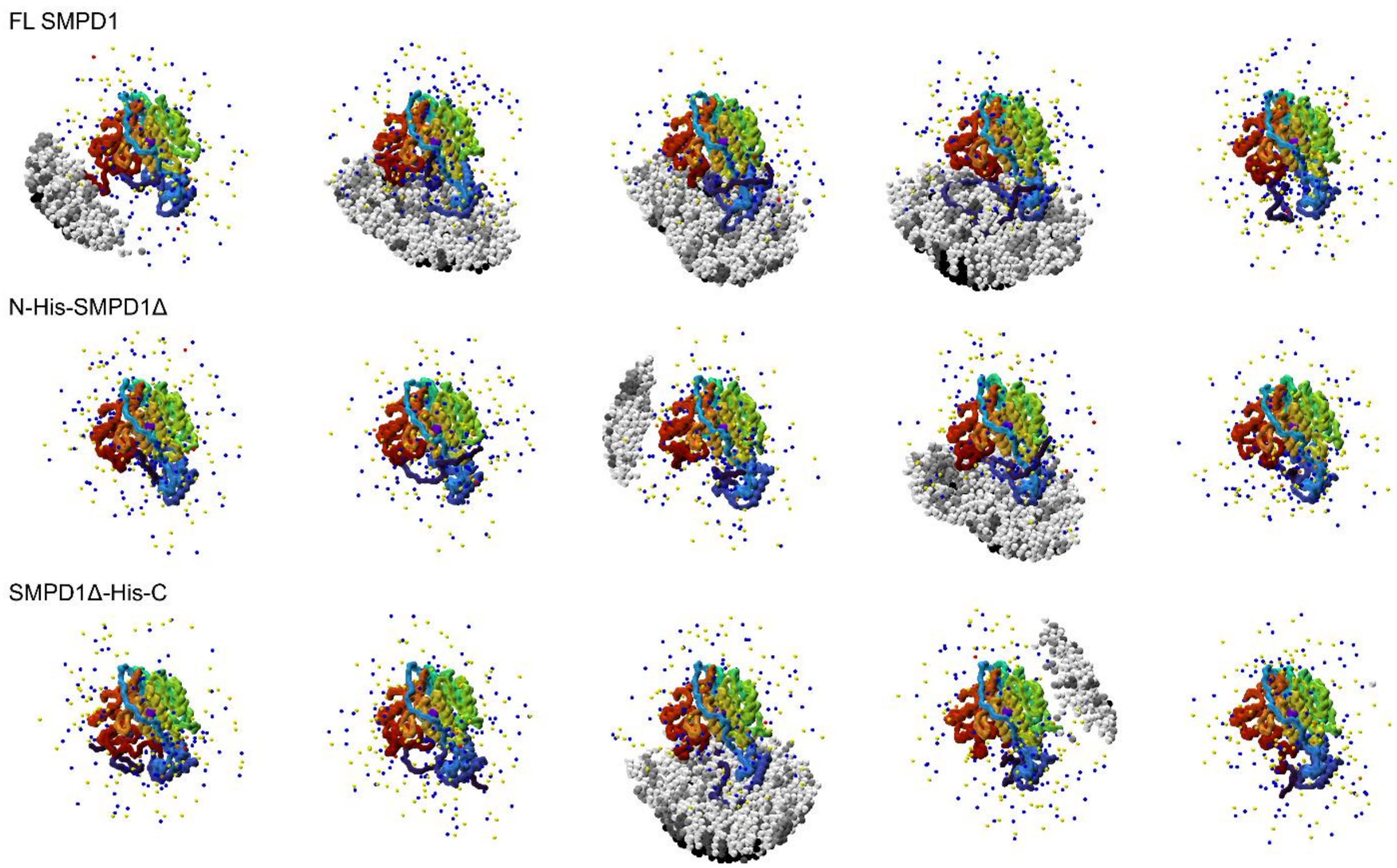

Supplemental Figure S8. Final frame snapshots of experimental ion systems. Images from left to right represent replicates.

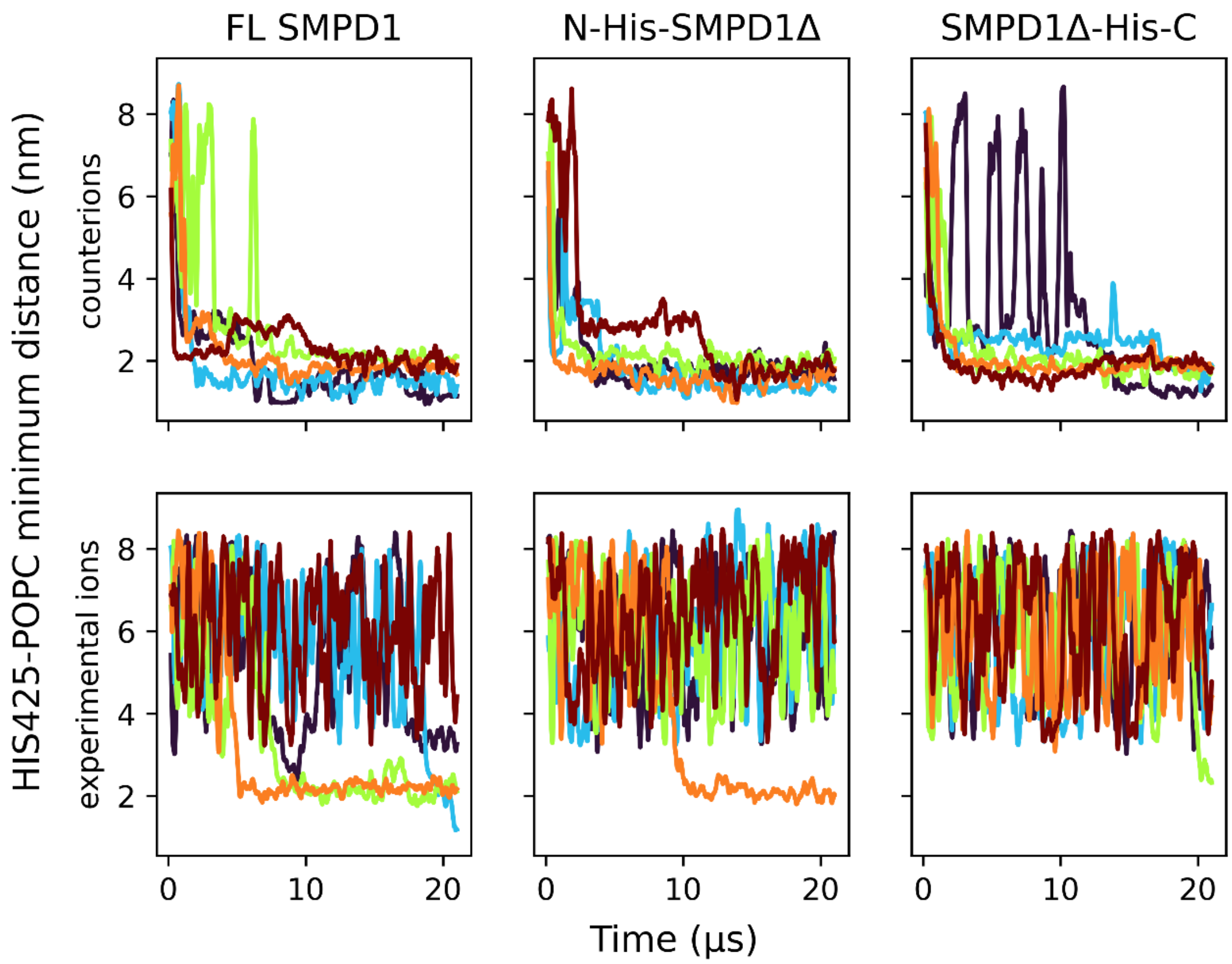

Supplemental Figure S9. Minimum distance between ASMase active site and POPC as a function of time. The system reaches the final binding position at ~2 nm. Colors represent replicates (n=5).

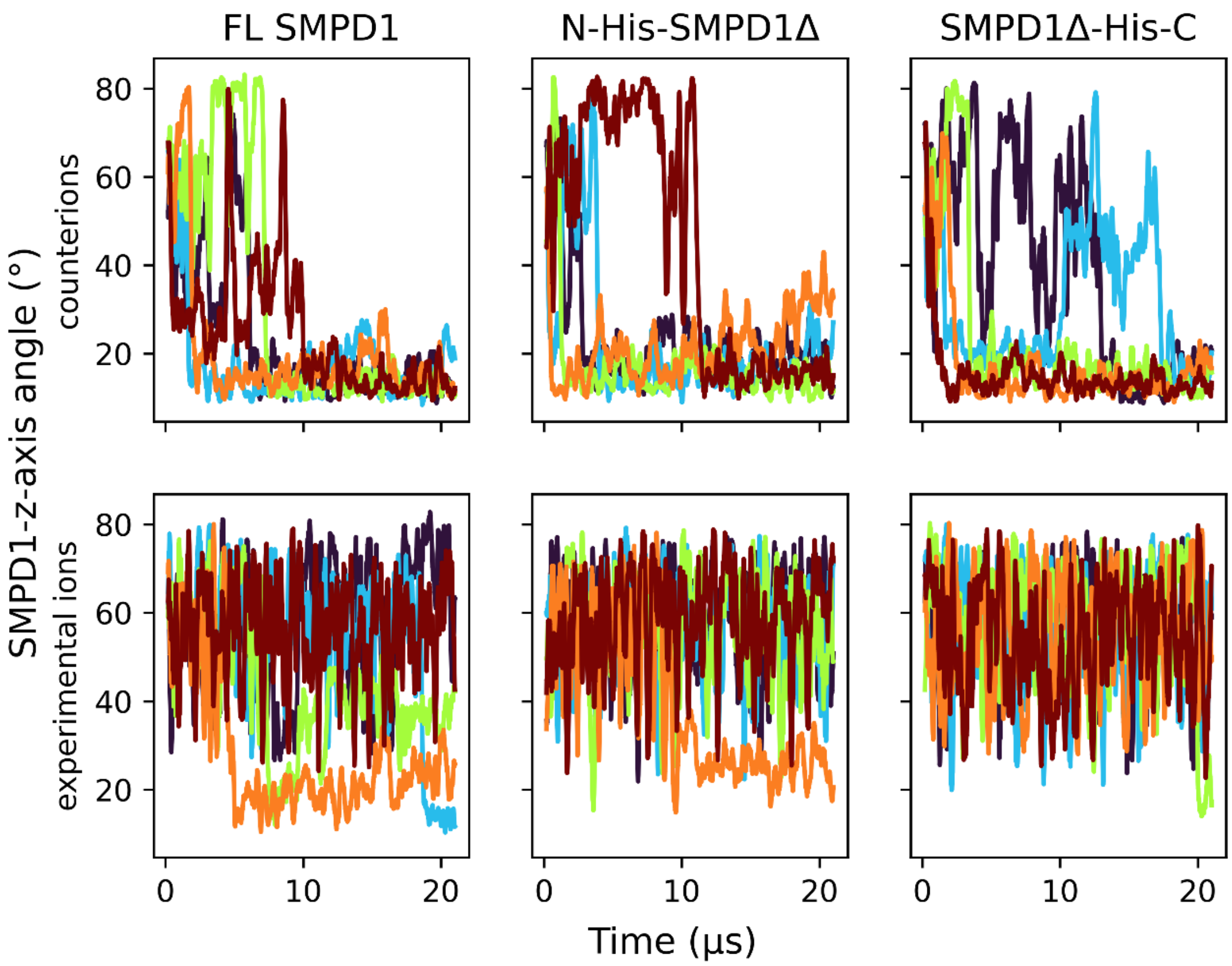

Supplemental Figure S10. The angle between ASMase HIS425 to GLY424 vector and Z-axis (lipid layer normal). A stabilized value indicates a state when system has found the final binding position.

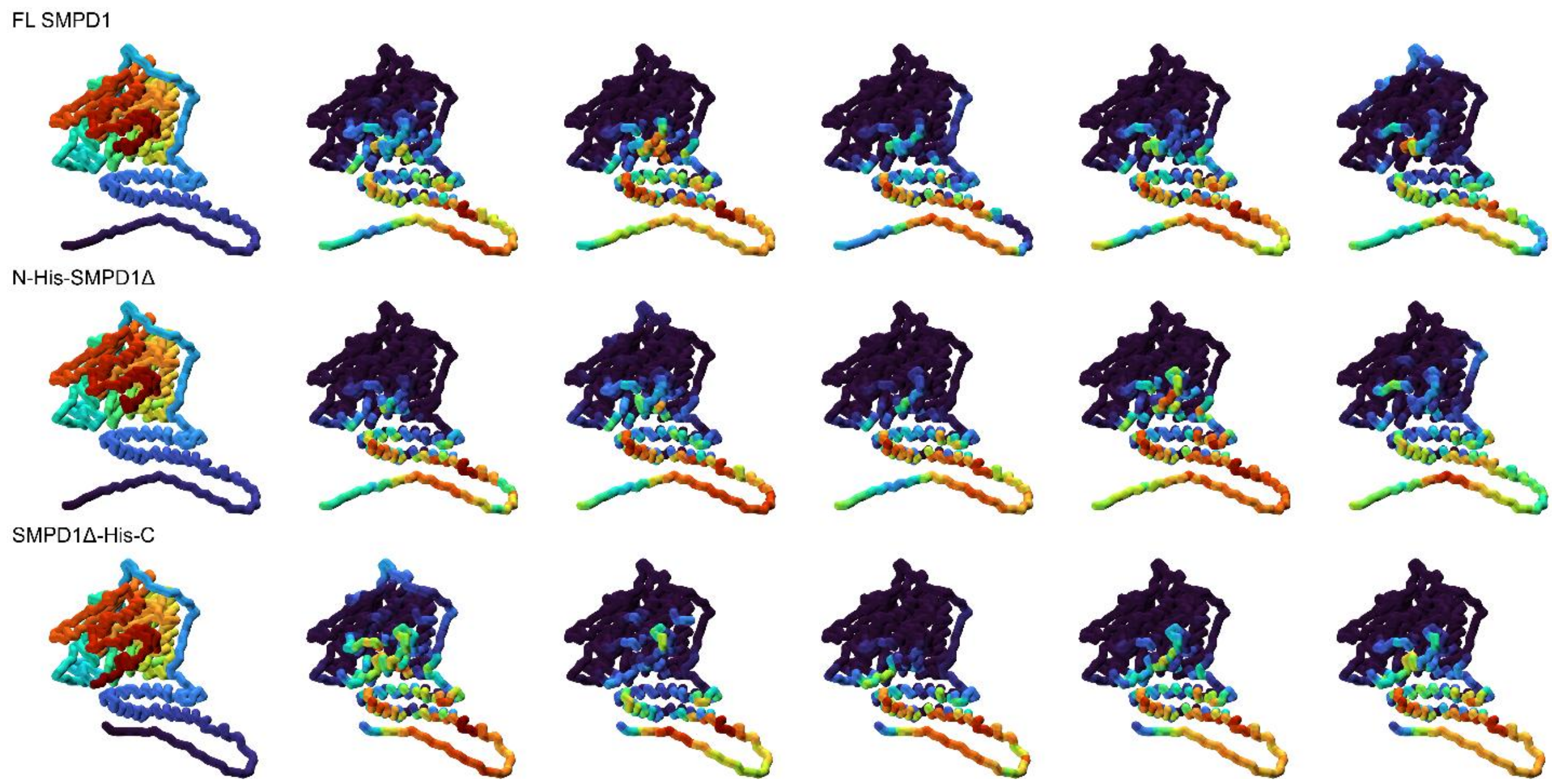

Supplemental Figure S11. Per residue average number of contacts to POPC per counterion system. The leftmost figure is colored per residue index from dark blue of N-terminus to dark red of C-terminus. Images from left to right represent replicates (n=5) with the greatest number of contacts in dark red to the least in dark blue. Coloring normalized per system.

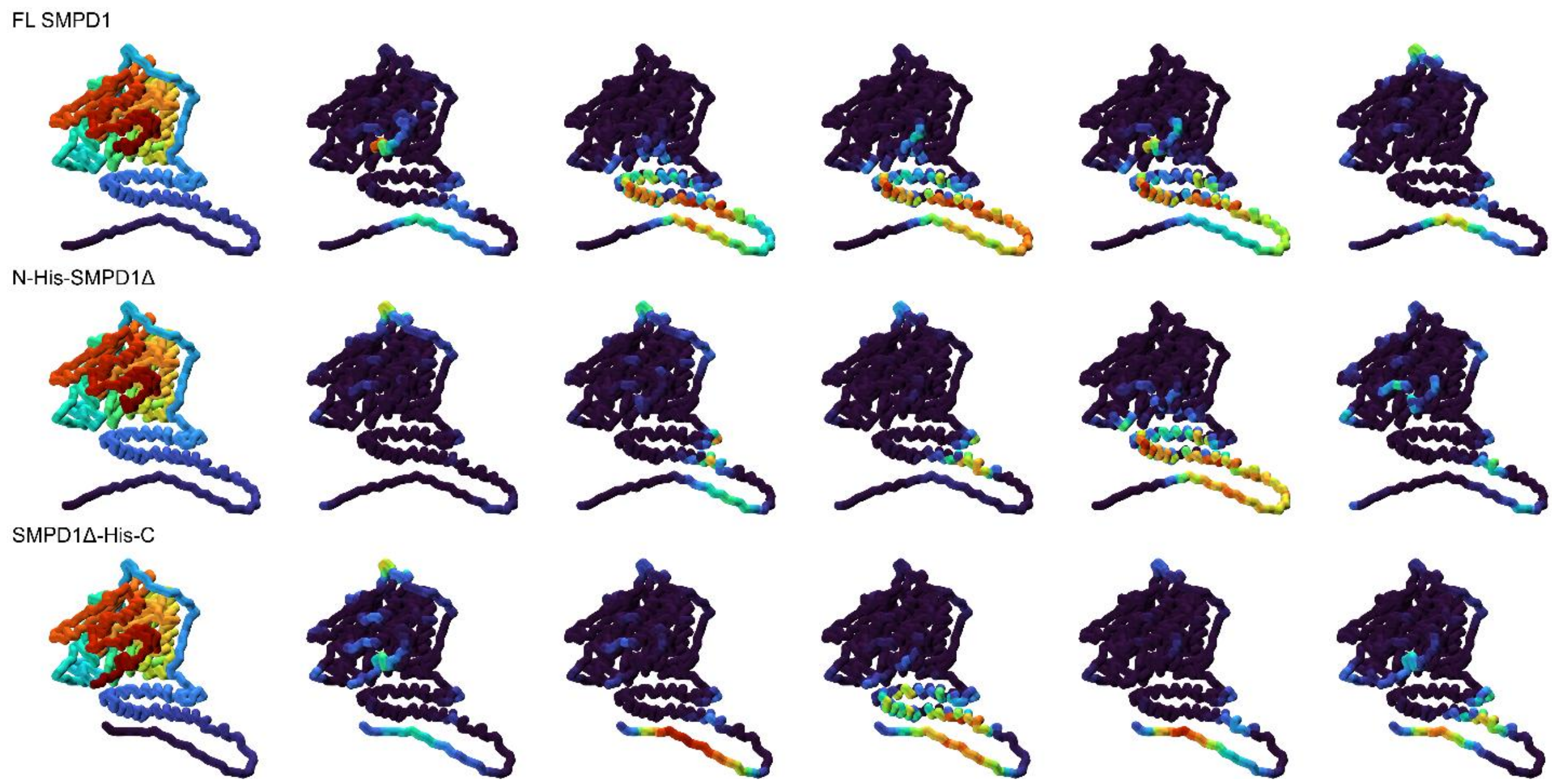

Supplemental Figure S12. Per residue average number of contacts to POPC per experimental ion system. The leftmost figure is colored per residue index from dark blue of N-terminus to dark red of C-terminus. Images from left to right represent replicates (n=5) with the greatest number of contacts in dark red to the least in dark blue. Coloring normalized per system.

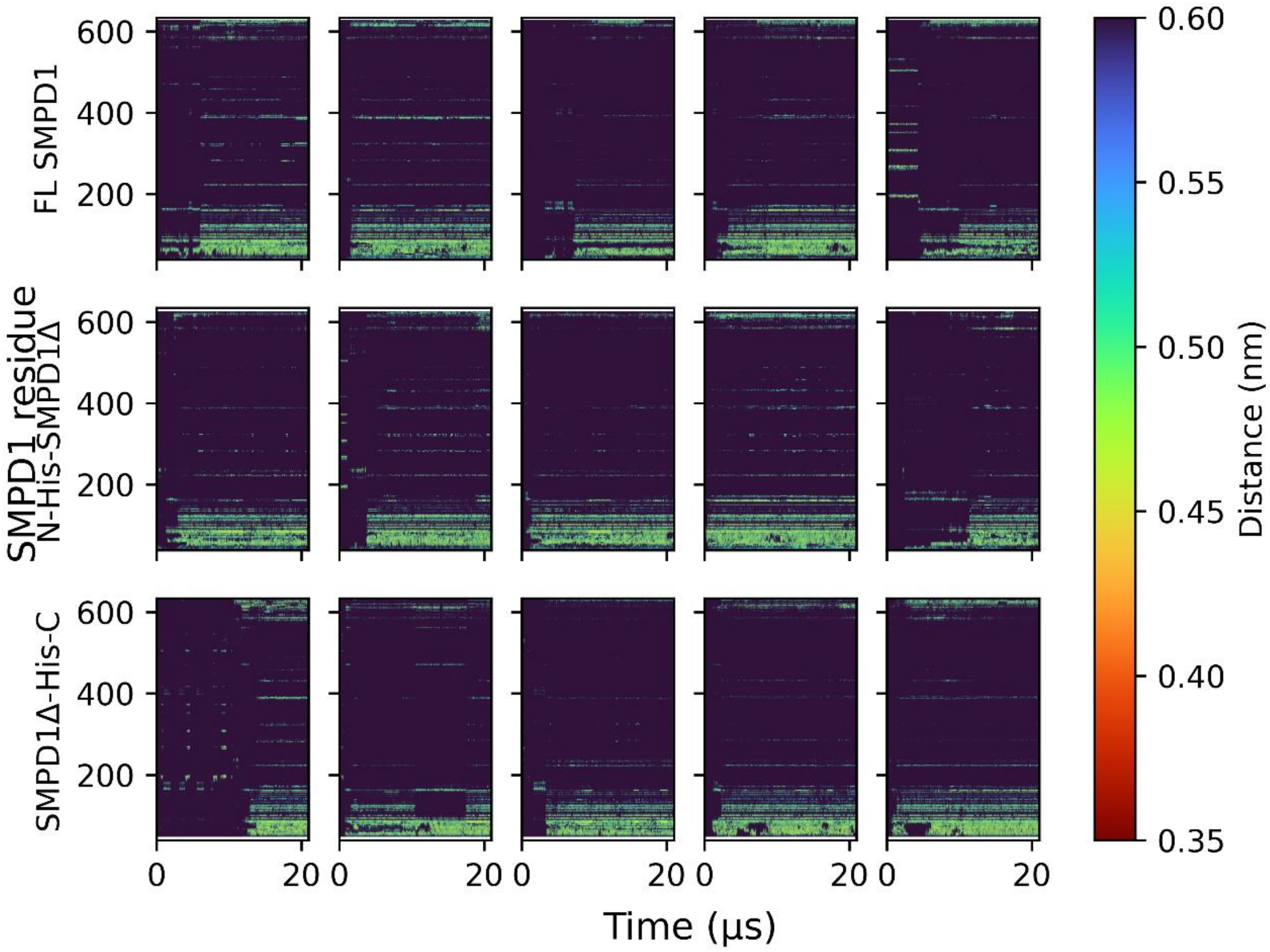

Supplemental Figure S13. Per residue minimum distance to POPC as a function of time per counterion system.

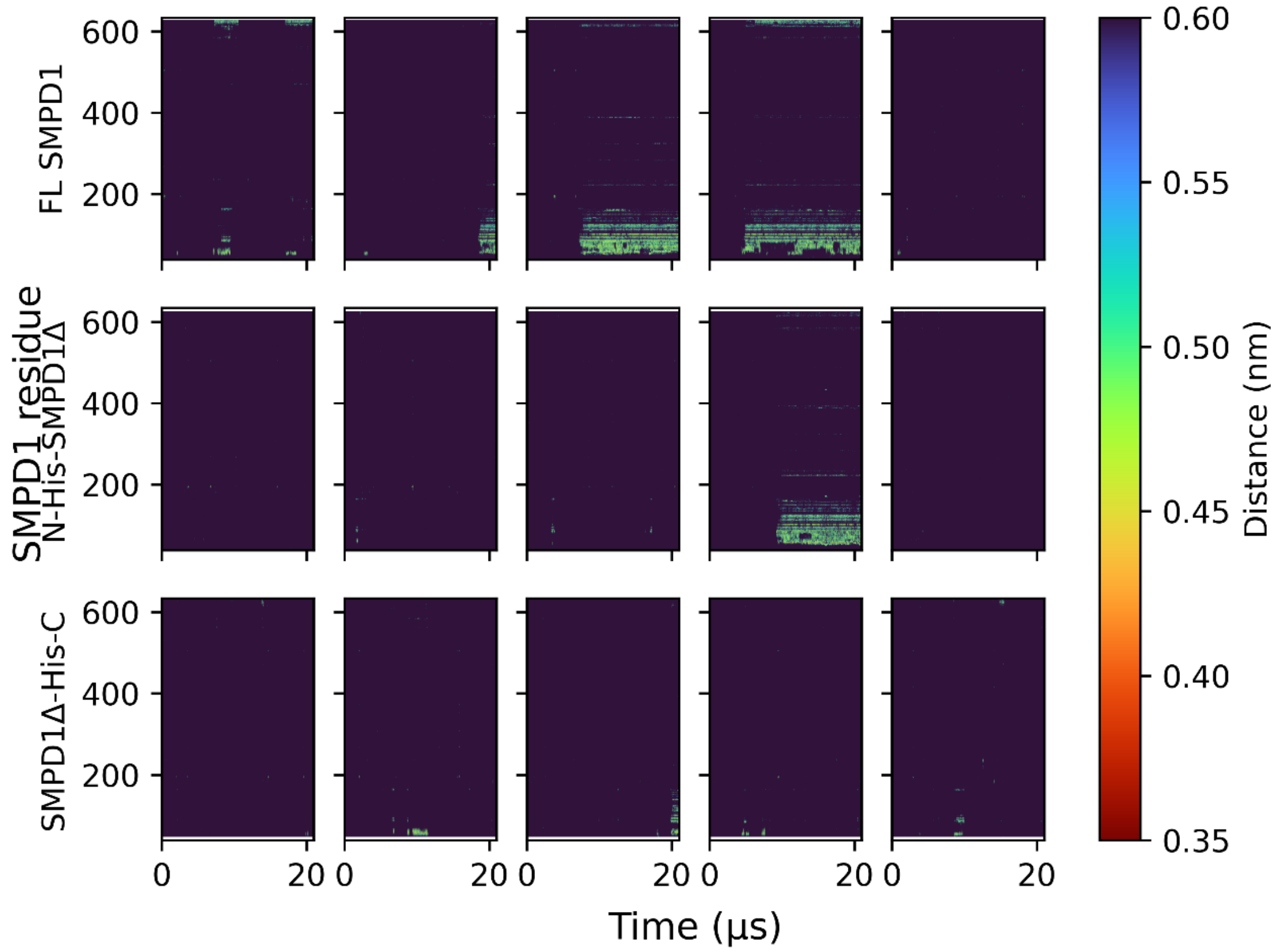

Supplemental Figure S14. Per residue minimum distance to POPC as a function of time per experimental ion system.

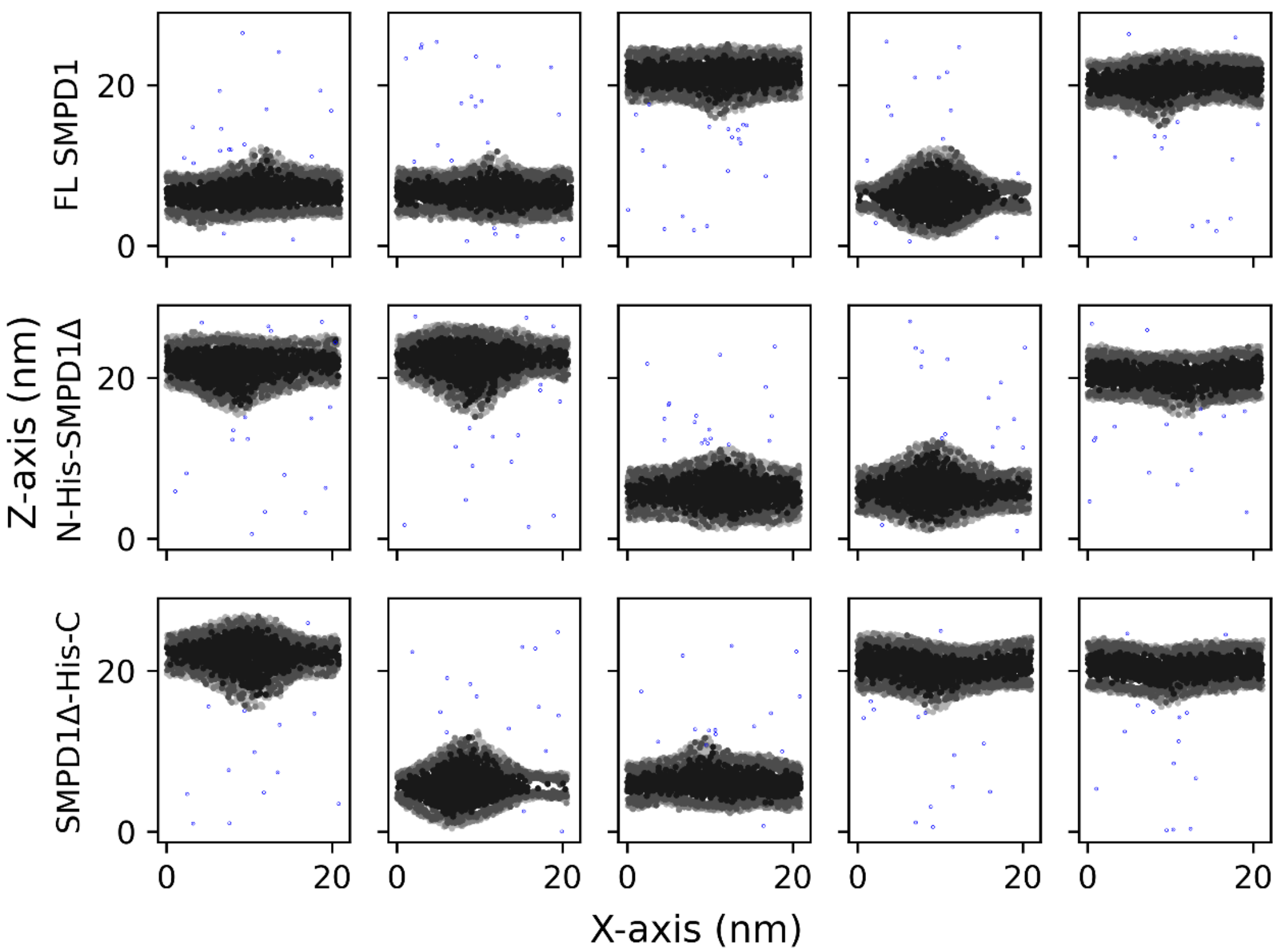

Supplemental Figure S15. Final frame snapshot of the lipids in counterion systems. ASMase is not shown in the image but is located at the center at x=~10, z=~15.

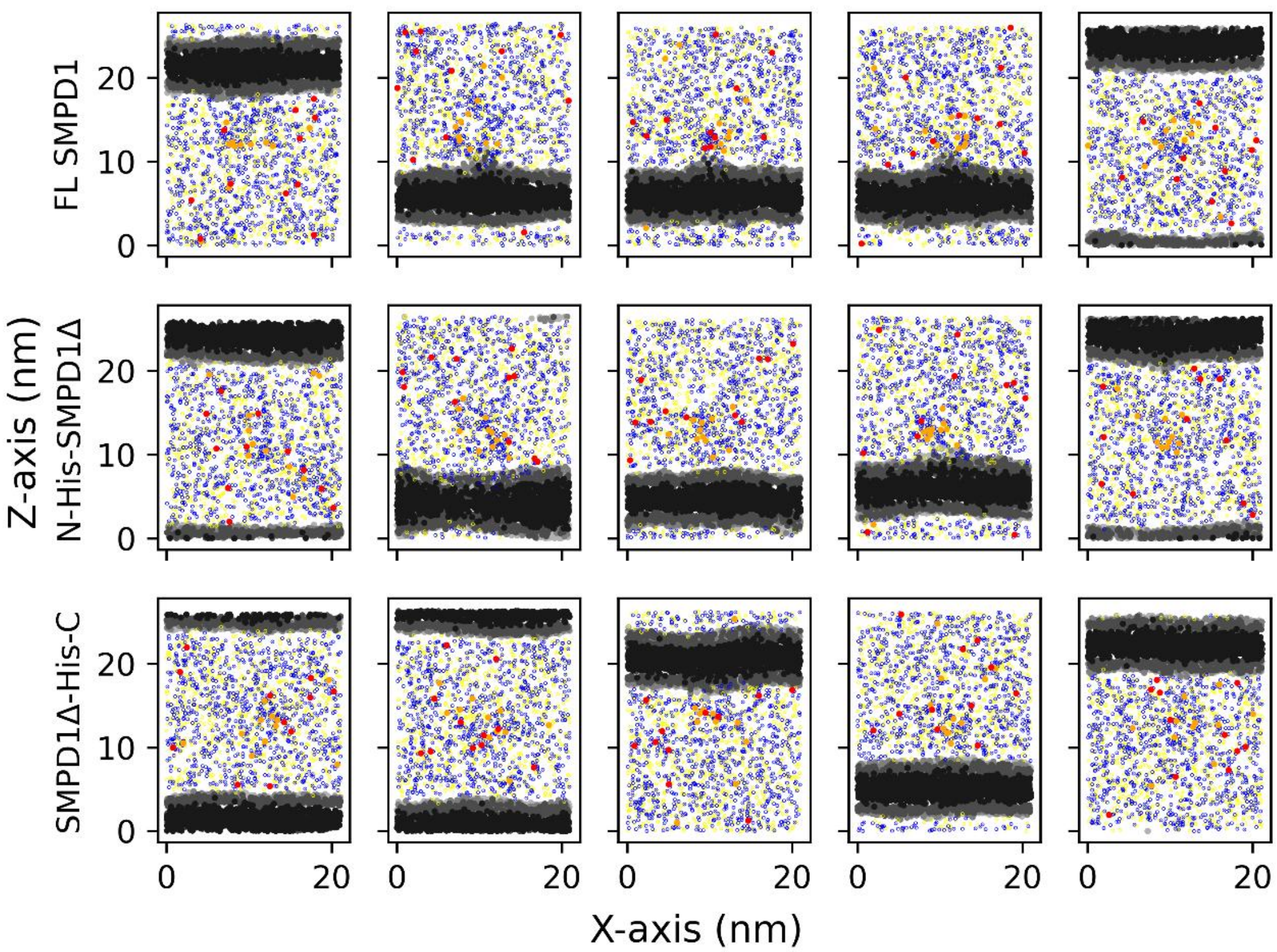

Supplemental Figure S16. Final frame snapshot of the lipids in experimental ion systems.

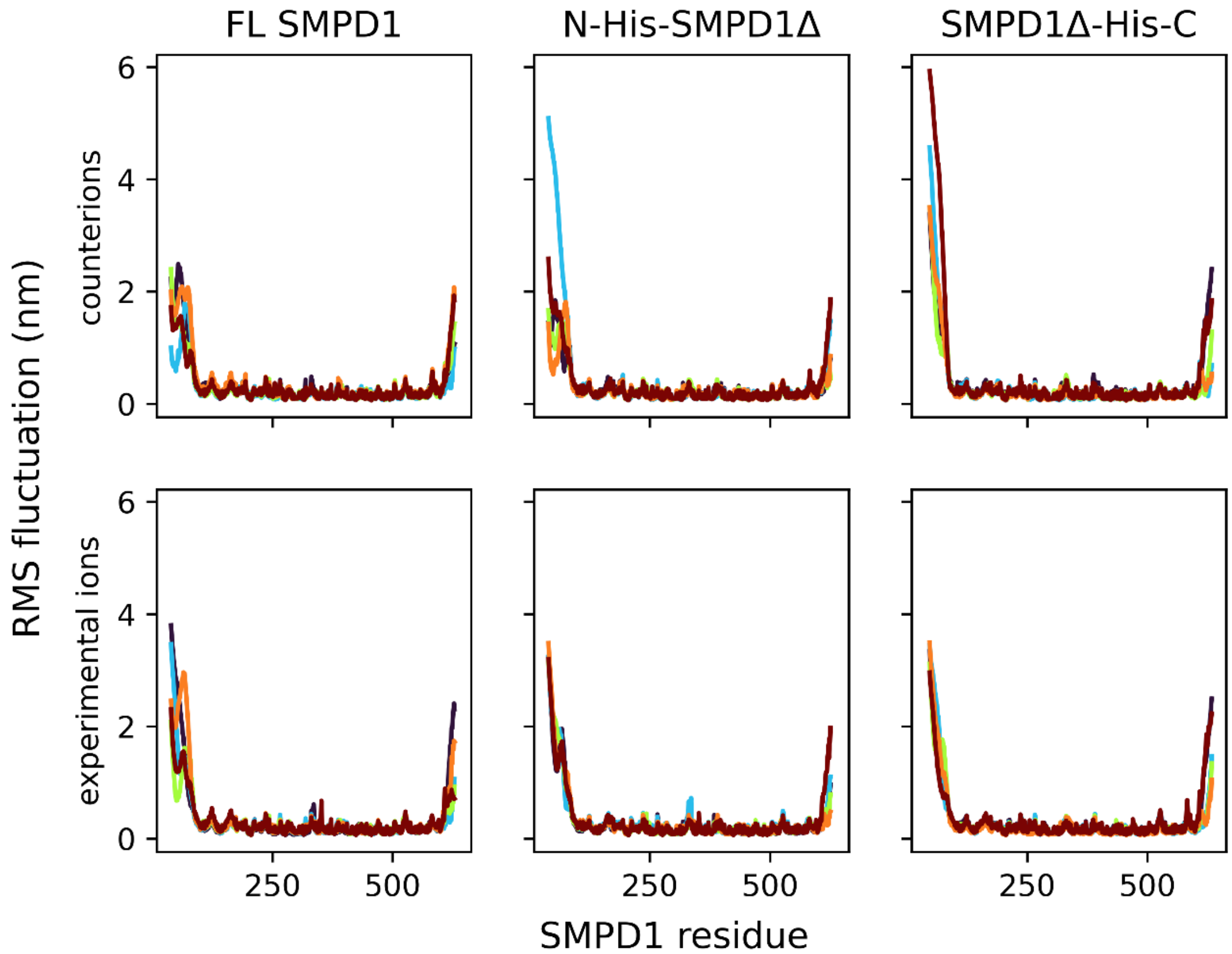

Supplemental Figure S17. RMSF profiles per system.

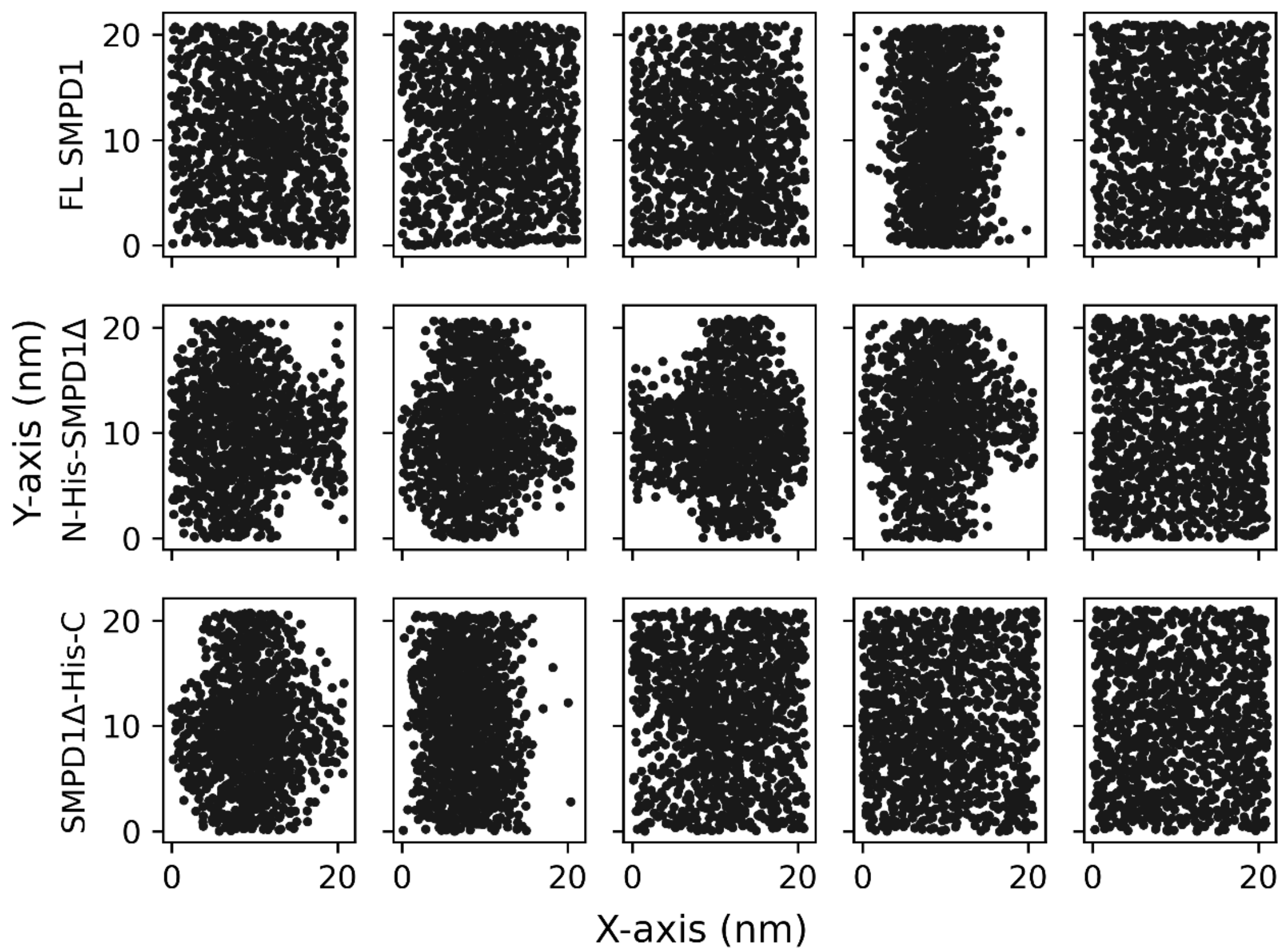

Supplemental Figure S18. Final frame snapshots of CHYO centers of mass in counterion systems.

ASMase is not shown in the image but is located at the center at x=~10, y=~10.

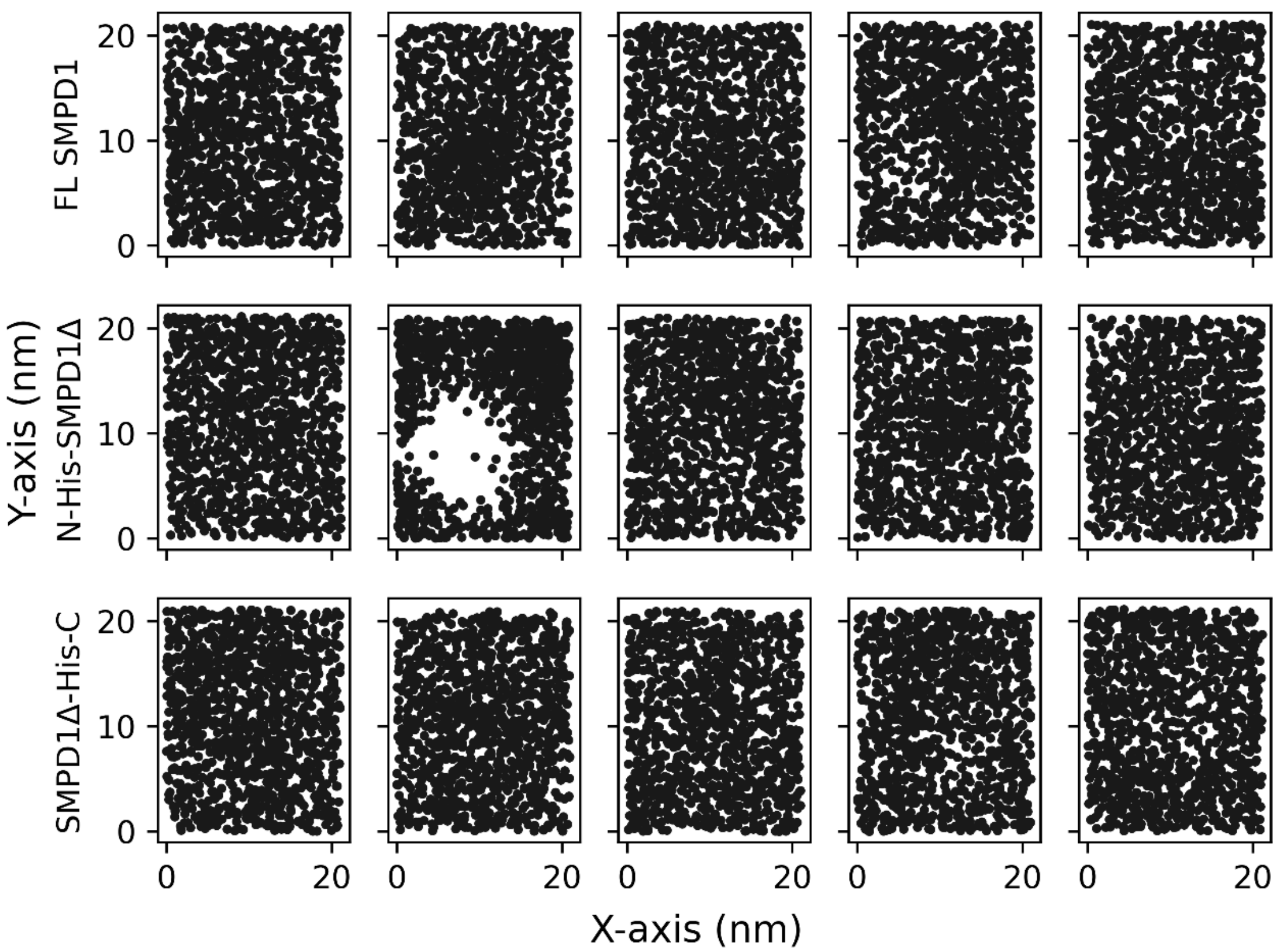

Supplemental Figure S19. Final frame snapshots of CHYO centers of mass in experimental ion systems.

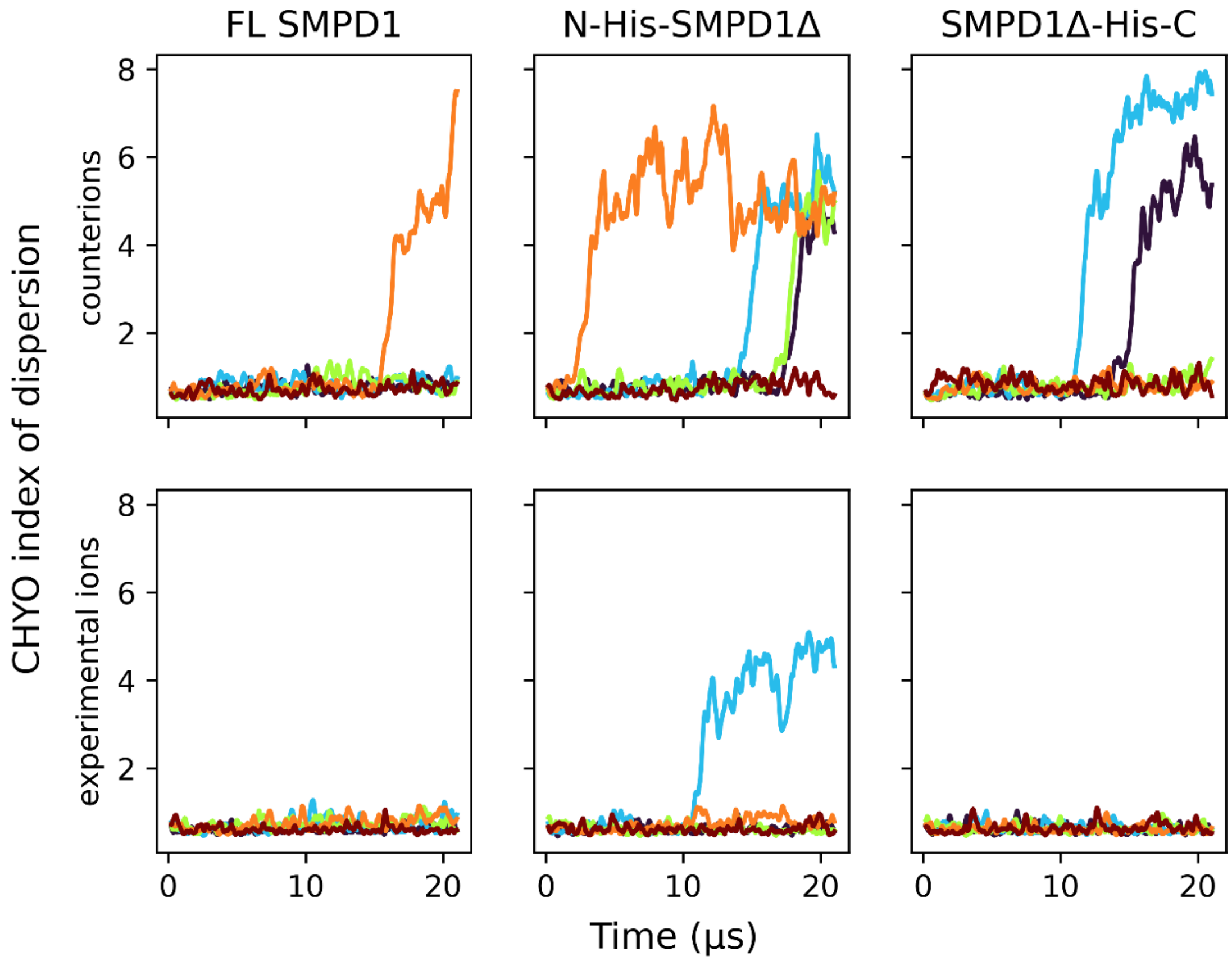

Supplemental Figure S20. Index of dispersion of cholesteryl oleate (CHYO) centers of mass on the XY-plane as a function of time. In counterion systems there is a higher tendency for the CHYO layer to aggregate. The trilayer was originally equilibrated in a system with no ions for 12 µs, so it is possible that a longer equilibration would have triggered the aggregation as well. In replicate 2 of experimental ions with *N-His-SMPD1Δ*, the aggregation occurred as well, and in that replicate ASMase did not bind to the membrane, which suggests this is independent of the disruption ASMase causes in the surface lipids, and ions simply stabilize the trilayer. However, we think it is likely that the disruption via ASMase binding is at least a catalytic factor. Furthermore, it is interesting that in counterion systems the aggregation centralizes to where ASMase is bound, as seen in Fig. S15-16 and S18-19, while the opposite occurs in the experimental ion system. Regardless, this artifact could likely be avoided by adjusting the barostat or the number of CHYOs.

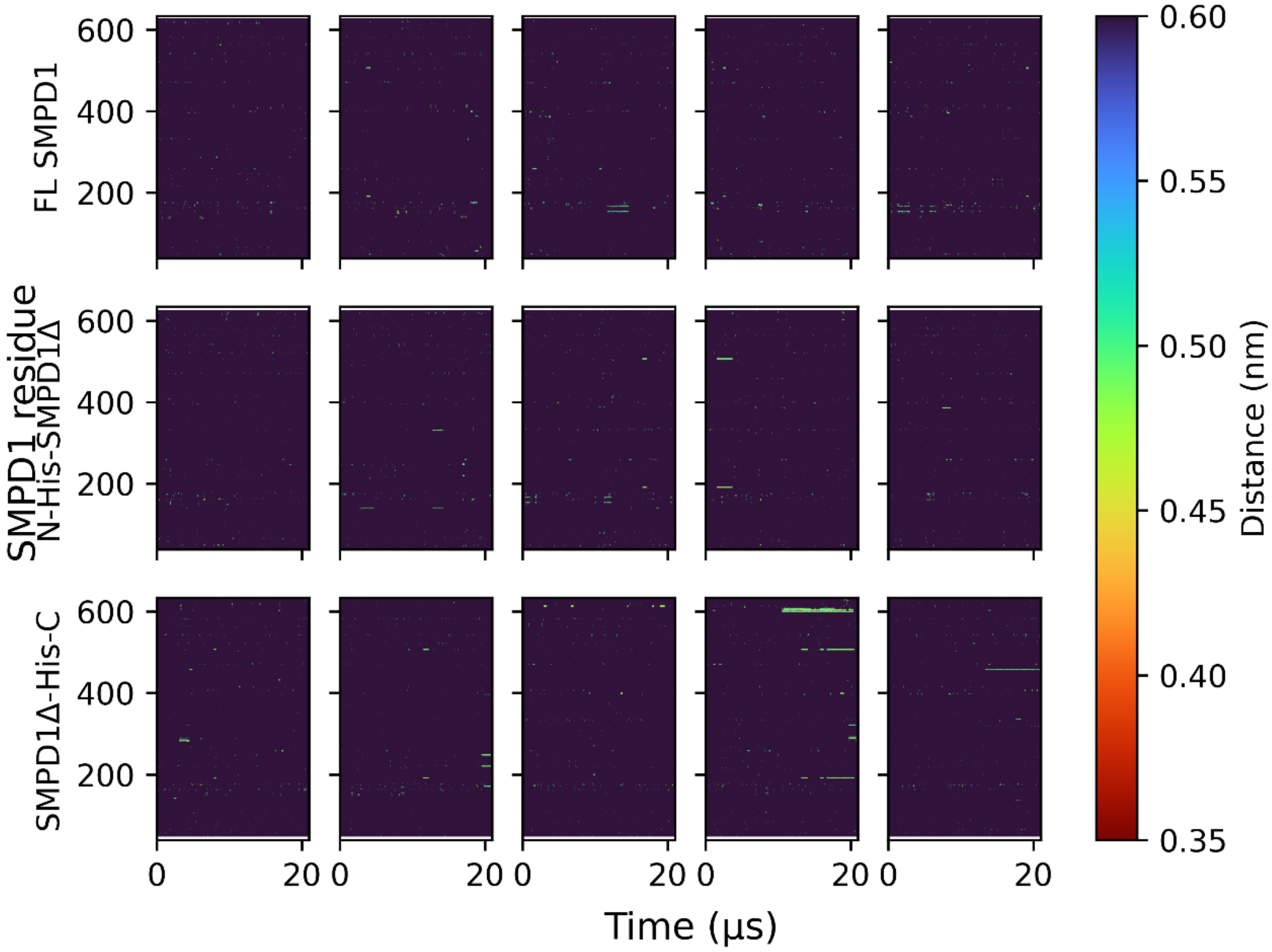

Supplemental Figure S21. Per residue minimum distance to calcium beads as a function of time per experimental ion system.

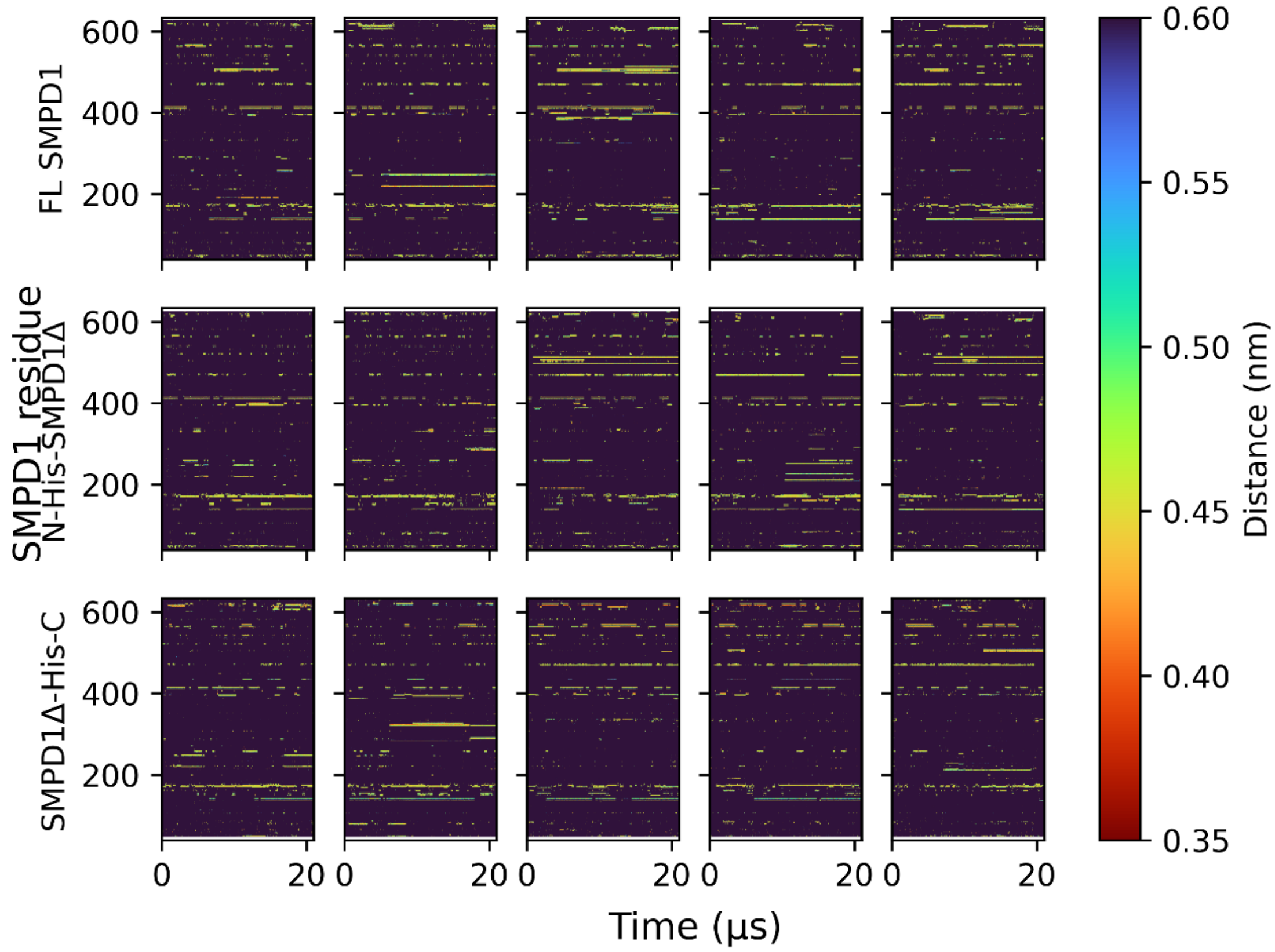

Supplemental Figure S22. Per residue minimum distance to magnesium beads as a function of time per experimental ion system. The only difference between calcium and magnesium beads in the Martini 3 force field is size: calcium is a small bead while magnesium is a tiny bead. We hypothesize that the primary reason for the reduced contacts is that calcium is not able to tunnel into ASMase between the protein chains, and thus it does enjoy long binding events. Zinc is necessary for ASMase activity, and it would be most accurately represented by a tiny bead like magnesium, but these binding events did not occur in the active site to a relevant capacity and thus are not specific to ASMase. Accurate modeling of ASMase with zinc bound with the Martini 3 force field would require an artificial bond.

A

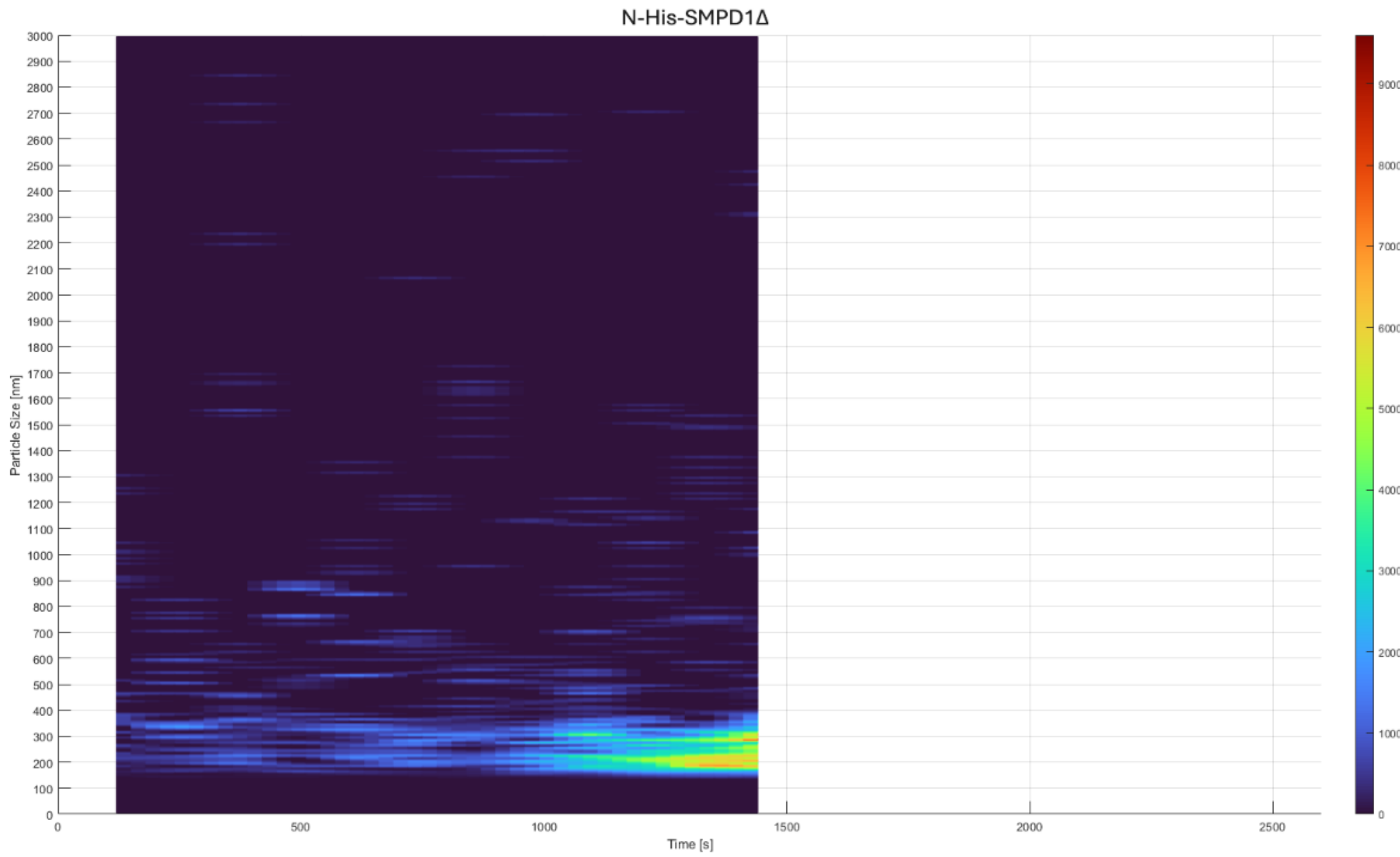
N-His-SMPD1Δ
Particle Size [nm]
Time [s]

B

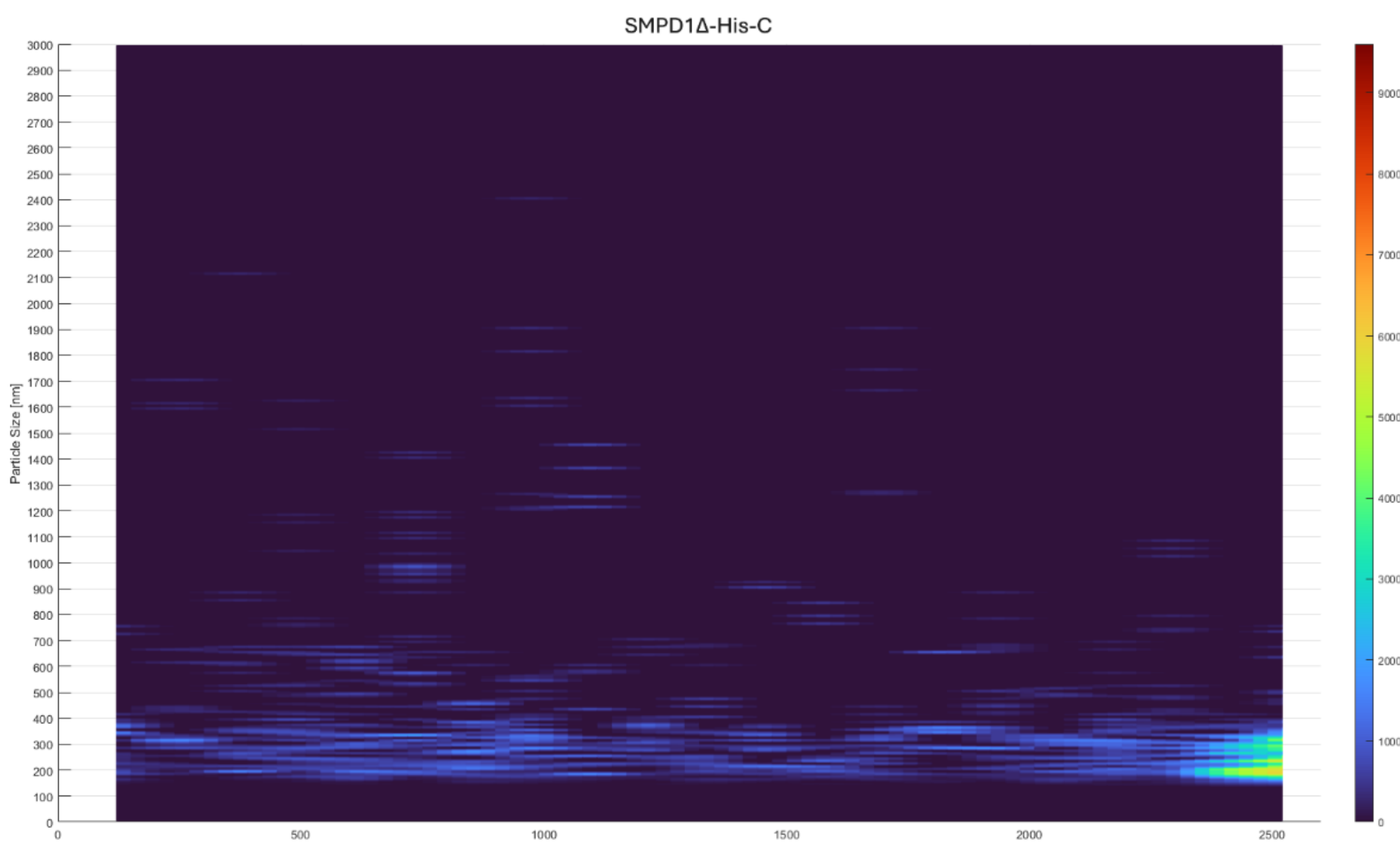
SMPD1Δ-His-C
Particle Size [nm]
Time [s]

C

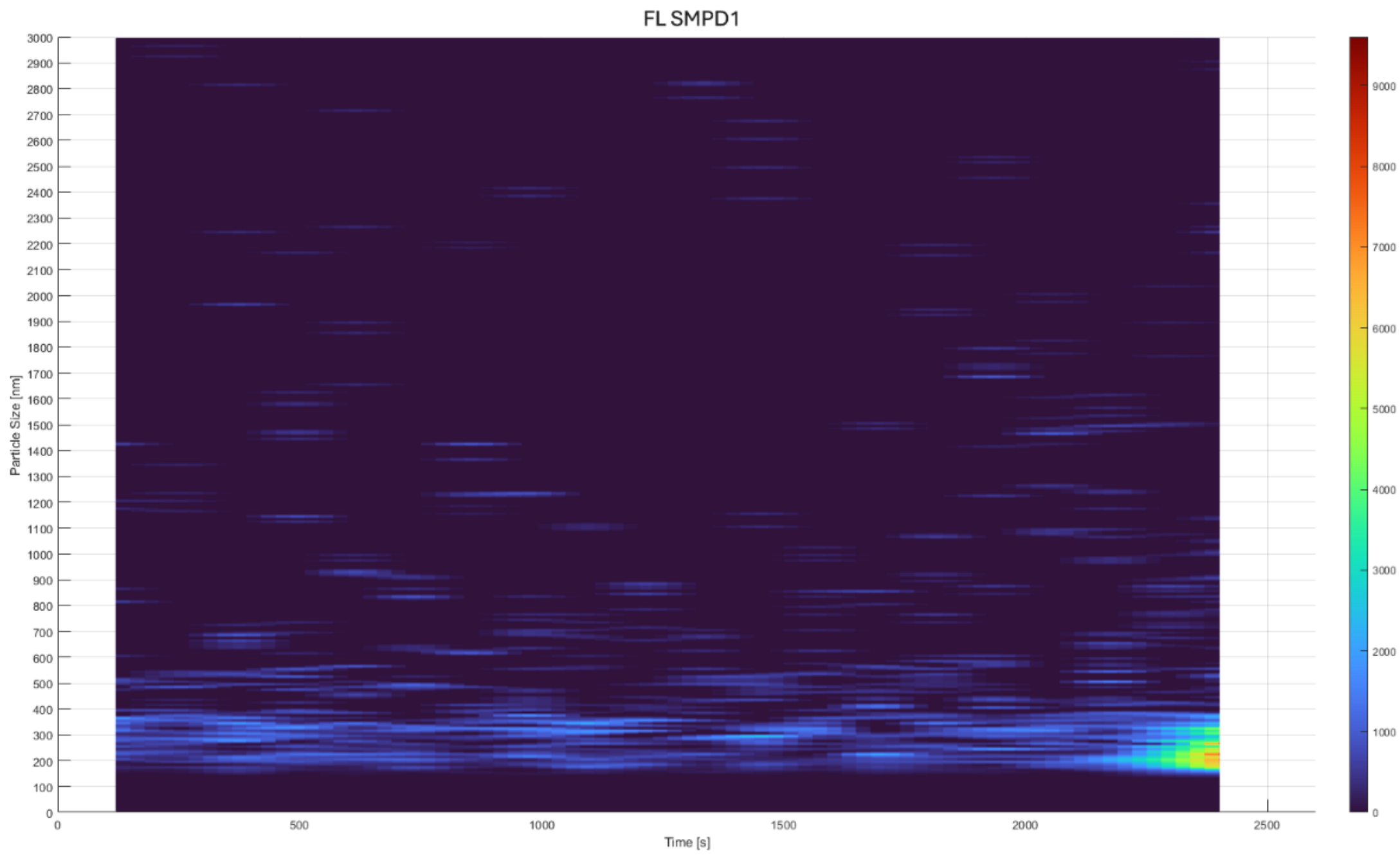

Supplemental Figure S23. Top view of the OF2i measurement results of rhASMase-induced LDL aggregation. The aggregation of LDL (0.01 mg/ml) was induced by rhASMase variants (0.03 mg/ml) in 20 mM MES pH 5.5, 150 mM NaCl, 2 mM MgCl2, 2 mM CaCl2 and 0.2 mM ZnCl2 at 37°C. The plotted values represent particle size (single-particle-based radius of hydration) and particle concentration on the gradient scale (particle number/ml). (A) *N-His-SMPD1Δ*; (B) *SMPD1Δ-His-C*; (C) *FL SMPD1*.

Supplemental Methods S1. Molecular Dynamics simulation setup.

Initial coordinates for rhASMases were generated with ColabFold version 1.6.1 with default settings and by using PDB 5I81 as a template (22, 23). Initially five conformations for each were generated, but since one structure of *FL SMPD1* had a closed saposin domain we generated ten for it instead and after ranking by pLDDT every other structure was used. This afforded a set with a roughly equal distribution of pLDDT values as *N-His-SMPD1Δ* and *SMPD1Δ-His-C*. The structures were refined with Schrödinger Maestro software package (version 14.3.129, release 2025-1). They were first aligned to 5I81 and then the zinc ions of 5I81 were added. The complexes were then processed with the Protein Preparation Workflow using pH 5.5 and otherwise default settings (24). The structures were exported and coarse-grained with Martinize2 version 0.15.0 using the Martini3-IDP force field with default GōMartini settings and by defining residues 39 to 87 as an IDR region (25-27). DSSP of MDTraj was used to define the secondary structure (28). The IDR region was defined because in the attained AlphaFold2 structures the N-terminus had an extended random coil conformation. Some presented with an additional alpha helix, but they were overridden for consistency.

A trilayer lipid system mimicking LDL surface composition was prepared manually (2). It contains a 1000 cholesteryl oleate (CHYO) layer surrounded by two phospholipid-cholesterol layers. Each phospholipid-cholesterol layer contains 470 palmitoyloleoylphosphatidylcholine (POPC), 180 palmitoylsphingomyelin (PSM) and 350 cholesterol (CHOL). POPC, PSM and CHOL parameters for Martini 3 force field were attained from CHARMM-GUI (29-32). CHYO parameters were constructed naively by combining Martini3 cholesterol and triolein parameters with Martini2 CHYO design principles (33). ASMase was not expected to interact with CHYO so the parameters were not validated beyond confirming that they qualitatively behave as expected. The trilayer was equilibrated for 12 µs using the default simulation parameters described below.

Once an equilibrated trilayer was attained, which had a size of ~21.0 nm in X- and Y-directions, the box size in the Z-direction was increased to 28.0 nm. ASMase was placed to the farthest point from the trilayer

(14 nm away under XYZ periodic boundary conditions). For experimental ion systems 150 mM NaCl, 2 mM $MgCl_2$ and 2 mM $CaCl_2$ were added, resulting in 834 sodium, 11 magnesium and 11 calcium ions. Zinc was omitted as in Martini 3 it would practically be represented by an additional magnesium ion. 878 chloride ions were added and ~20 to neutralize the system. For counterion systems only neutralizing chloride ions were added. For each condition (three rhASMases and two ion configurations) five replicates were run with the different parameters attained from AlphaFold2 generated conformations, resulting it 30 systems in total. After energy minimization and a 1 ns equilibration with a 10 fs timestep, production systems were run for 21 μs with a 20 fs timestep. Coordinates were saved every ns without water. Short-range neighborlist cut-off was 1.35 nm, Van der Waals were handled with a 1.1 nm cut-off and electrostatics with a reaction-field cut-off of 1.1 nm and a relative dielectric constant of 15 and infinity for the reaction field. A V-rescale thermostat was set to 310 K, and a C-rescale semi-isotropic barostat to 1 bar (35, 36). Constraints were handled with LINCS with a highest order of 8 and number of iterations of 2 (37). Simulations were prepared with the GROMACS software package version 2024.3 and production systems run with version 2025.4 (34). Minimum distances were calculated with gmx mindist, contacts with gmx pairdist with a 0.6 nm cut-off, Root mean square fluctuations (RMSF) with gmx rmsf averaged per residue. For LEU47-LEU87 distance, HIS425-GLY424 to Z-axis angle analysis and index of dispersion custom scripts were written with MDAnalysis (72). For index of dispersion the XY-plane was split to 100 bins and the number of CHYO centers of mass per bin were calculated. For visualization of time dependent data running averages were calculated with a 200 ns window. VMD was used for renders (73).
